# Fecal microbiota transplantation alleviates adverse pregnancy outcomes and intestinal injury in experimental malaria

**DOI:** 10.1371/journal.ppat.1014459

**Published:** 2026-08-20

**Authors:** Su Han, Jie Wan, Rongrong Dai, Weizhong Kong, Zhenli Xu, Yu Zhang, Rui Chen, Yang Cheng, Yifan Sun

**Affiliations:** 1 Department of Public Health and Preventive Medicine, Wuxi School of Medicine, Jiangnan University, Wuxi, China; 2 Department of Laboratory Medicine, Affiliated Hospital of Jiangnan University, Wuxi, China; 3 Jinan Vocational College of Nursing, Jinan, China; 4 Affiliated Hospital of Jiangnan University, Wuxi, China; 5 Institute for Prevention and Control of Tropical Diseases and Chronic Diseases, Hainan Provincial Center for Disease Control and Prevention (Hainan Academy of Preventive Medicine), Haikou, China; University of Manchester, UNITED KINGDOM OF GREAT BRITAIN AND NORTHERN IRELAND

## Abstract

Malaria in pregnancy remains a major global health concern, contributing significantly to maternal and offspring morbidity and mortality. While gut microbiota dysregulation has been implicated in pregnancy complications and malaria pathogenesis, its functional role and the underlying mechanisms within the gut-placenta axis during placental malaria remain poorly understood. In pregnant mice, *Plasmodium berghei* ANKA infection disrupted the gut-placenta axis, leading to intestinal inflammation, placental injury and reduced fetal weight. Microbiome analysis revealed gut dysbiosis characterized by reduced abundance of *Ligilactobacillus* and increased abundance of *Desulfovibrio*. The metabolomic profiling identified disruption in amino acid and fatty acid metabolism, including changes in metabolites such as indole-3-propionic acid and taurine. These microbial and metabolic alterations may contribute to impaired intestinal barrier integrity and dysregulated inflammatory responses. Importantly, fecal microbiota transplantation (FMT) restored gut microbial balance, alleviated colonic and placental inflammation, and improved offspring growth. These findings provide novel mechanistic insights into the gut-placenta axis in malaria during pregnancy. Future studies should validate these findings in clinical settings and explore alternative microbiota-targeted interventions.

## Author summary

Malaria in pregnancy remains a major global health concern, contributing significantly to maternal and offspring morbidity and mortality. While gut microbiota dysregulation has been implicated in pregnancy complications and malaria pathogenesis, its functional role and the underlying mechanisms within the gut-placenta axis during placental malaria remain poorly understood. We found that malaria infection disrupted gut microbial homeostasis, characterized by reduced *Ligilactobacillus* and increased *Desulfovibrio*, and was accompanied by impaired intestinal barrier integrity, metabolic dysregulation, and placental inflammation. Together, these disruptions along the gut-placenta axis may contribute to impaired offspring growth. Importantly, fecal microbiota transplantation from healthy uninfected donors to infected mothers restored microbial balance, alleviated intestinal and placental inflammation, and partially improved offspring outcomes. Our work provides mechanistic insights linking the gut microbiome to pregnancy complications in malaria and points to microbiome-targeted strategies as potential future avenues for intervention. Further studies are needed to validate these findings in clinical settings.

## Background

Malaria, caused by parasites of the genus Plasmodium, is one of the most prevalent infectious diseases in tropical and subtropical regions. It is estimated that there were 263 million clinical cases and approximately 597,000 deaths in 2023 [1]. In sub-Saharan Africa, where the highest burden of malaria is concentrated, *P. falciparum* is the primary cause of disease and death. Each year, millions of pregnant women are at risk of acquiring *P. falciparum* infections, posing a significant threat to maternal and neonatal health [1]. Maternal malaria infection profoundly impacts the postnatal health and survival of infants [2,3]. Malaria-associated low birth weight is estimated to have a fatality rate of 37.5% [4], and infants born to malaria-infected women are more susceptible to malaria in early life [5,6]. Despite progress in prevention and treatment, adverse pregnancy outcomes related to malaria remain high [1]. Therefore, understanding the pathogenesis of malaria during pregnancy is critical for reducing associated maternal and offspring morbidity and mortality in malaria-endemic regions.

Beyond its classic systemic manifestations, malaria in pregnancy frequently exhibits gastrointestinal symptoms such as nausea, vomiting, abdominal pain, and diarrhea. Accumulating evidence indicates that Plasmodium infections disrupt intestinal barrier integrity through microvascular sequestration of infected erythrocytes, local inflammatory activation, and subsequent disruption of epithelial tight junctions [7–11]. This impairment increases the risk of bacterial translocation into the bloodstream, potentially causing concurrent bacteraemia and sepsis, particularly in severe cases involving children in Africa [12]. Furthermore, elevated plasma concentrations of bacterial metabolites have been reported in patients with severe falciparum malaria [13]. These observations highlight the potential interplay between intestinal injury and malaria infection in the pathogenesis of the disease.

The gut microbiota, comprising diverse microbial communities in the gastrointestinal tract, plays a critical role in maintaining intestinal barrier integrity and regulating immune responses [14,15]. Recent research has indicated that imbalances or dysregulation of gut microbiota are associated with exacerbated inflammation and increased susceptibility to infections during malaria [16,17]. However, existing studies on the gut microbiota in malaria have primarily focused on non-pregnant models or clinical observations of microbial shifts. Consequently, the functional implications of gut microbiota homeostasis during *Plasmodium* infection in pregnant hosts remain- poorly understood.

Gut microbiota dysregulation has been implicated in pregnancy complications and adverse offspring outcomes through the gut-placenta axis [18]. Smith et al. [2] demonstrated that gut microbiota composition was associated with both susceptibility to malaria during pregnancy and fetal outcomes, and further identified specific microbial taxa related to infection outcomes. However, how the gut microbiota influences offspring development, intestinal integrity, and host metabolism through the gut-placenta axis remains incompletely elucidated.

In this study, pregnant mice were intravenously infected with *Plasmodium berghei ANKA (Pb*A*),* resulting in placental damage, intestinal inflammation, adverse pregnancy outcomes and disruption of the gut-placenta axis. Gut microbiota composition in fecal samples was assessed using the Illumina MiSeq platform, and serum metabolite levels were analyzed through LC-MS/MS-based metabolomics. The functional role of the gut microbiota was further investigated through fecal microbiota transplantation (FMT) experiments. Additionally, we evaluated the transgenerational impact of maternal infection by assessing the growth and reinfection susceptibility of the offspring. Collectively, our findings provide insights into how the gut microbiota influences pregnancy outcomes by modulating intestinal barrier integrity, host metabolism, and inflammatory responses, thereby providing deeper mechanistic insights into gestational malaria pathogenesis.

## Materials and methods

### Ethics statement

The animals were maintained and used according to the Regulations for the Administration of Affairs Connecting Experimental Animals in China and the international research animal use guidelines. The protocol was approved by the Institutional Animal Care and Use Committee of the Jiangnan University (20220315i0361020), and the mice were housed in the Animal Center of College Laboratory.

### Gestation timing and pregnancy monitoring

Female BALB/c mice, aged eight to twelve weeks were obtained from Changzhou Kavins Laboratory Animal Co, Ltd. The mice were bred and maintained under specific-pathogen-free (SPF) conditions. Two to three females were put together with one male for 2 days, and examined for the presence of vaginal plugs every morning. The detection of the vaginal plugs and the measurement of body weight were jointly used to time gestation [19,20]. The day of finding the vaginal plug was considered as gestation day one (G1) and pregnancy progression was monitored every other day by weighing the females. Successful fertilization was confirmed between gestation days 10 and 13 (G10-G13), when the animals showed an average increase of 3–4 g in body weight. Thus, weight gain was taken as a sign of pregnancy and abrupt weight loss as an indicator of pregnancy damage or interruption.

### Infection with *Plasmodium berghei* ANKA

The *Pb*A strain was maintained through continuous passaging in our research facility at Jiangnan University. Cryopreserved *Pb*A-infected erythrocytes were retrieved from liquid nitrogen and immediately thawed in a 37°C water bath. Then the viable parasites were intraperitoneally inoculated into mice for experimental infection.

Based on the preliminary studies demonstrating that earlier administration (G1-10) resulted in pregnancy termination prior to offspring viability, the timing of infection was scheduled for gestational day 13 (G13) [20]. Regarding the infection doses, it was reported that lower inoculum doses (≤1 × 10^5^) failed to establish effective infection models, whereas higher doses (≥1 × 10^7^) induced acute lethal responses in hosts [21,22]. The dosage parameter (1 × 10^6^) was sufficient to sustain parasitemia and maintain the stability of the pregnancy model. Thus, pregnant mice received intravenous inoculation of 1 × 10^6^ PbA-infected erythrocytes at G13 and were assigned to three groups: PbD0, PbD3 and PbD5 post-infection (n = 6). Pregnant uninfected mice were assigned to the UN group and received an intravenous injection of 200μl PBS per 20 g body weight to control for handling (n = 10). To determine pregnancy-specific effects, a control cohort of non-pregnant mice was also infected with PbA and analyzed at 3 (non-pregnant-PbD3) and 5 (non-pregnant-PbD5) days post-infection (n = 6).

Peripheral blood samples were obtained through caudal venipuncture at 24-hour intervals for preparation of thin blood films, which underwent fixation in methanol followed by Giemsa staining (pH 7.2). Parasitemia quantification was performed through systematic microscopic examination using oil immersion optics (1000 × magnification). The pregnant females were subjected to caesarian section at G18 for fetal survival and placenta pathology observation.

### Antibiotic treatment and fecal microbiota transplantation

For depletion of the microbiota, mice were treated with a broad-spectrum antibiotic mixture (ABX) consisting of 100 mg/kg vancomycin, 200 mg/kg neomycin sulfate, 200 mg/kg metronidazole and 200 mg/kg ampicillin by oral gavage for 3 days from G8 to G11 in the infected mice (IN-AB group) [23]. A fresh antibiotic solution was prepared daily to ensure activity. Mice in the IN group received no antibiotic treated.

To prepare FMT material, fresh fecal samples from healthy gestation-phase BALB/c mice were collected daily into sterile cryovials. Aliquots (100 mg, 5–6 fecal pellets) were immediately placed in ice-cooled containers. Processing involved homogenization with chilled PBS (1:10 w/v), 15 min incubation at 4°C, and 70 μm membrane filtration. Centrifugation (4°C, 5 min) yielded bacterial pellets resuspended in PBS to 1 × 10^11^CFU/L for transplantation. Prepared inocula were delivered to murine recipients through standardized oral gavage procedures at a dosage of 10 mL/kg body weight utilizing precision feeding apparatus [24].

For FMT experiments, infected pregnant mice were randomly assigned to IN and IN-FMT groups, and FMT was performed daily from G13 to G18 in the IN-FMT group. To mitigate the potential risk of pathogen transmission during FMT and ensure experimental reliability, the following quality control measures were implemented.All donor mice were sourced from a SPF-grade facility and were pre-screened for common murine pathogens, and only healthy individuals without signs of gastrointestinal infection were selected; the prepared bacterial suspensions were tested via both aerobic and anaerobic culture prior to transplantation to confirm the absence of exogenous pathogenic contamination; all procedures were performed within a biosafety cabinet using sterile instruments to avoid cross-contamination [25,26]. All mice were maintained under identical feeding and environmental conditions to ensure consistency. Their body weights were monitored weekly throughout the treatment period. As a vehicle control, mice in the IN group were gavaged with PBS. The experimental timeline is summarized in S1 Fig.

### Assessment of pathological changes in the placenta and colonic tissue

Placenta and colonic tissue were processed according to standard protocols (Servicebio), including fixation, embedding, paraffin sectioning, and cryosectioning. Paraffin-embedded sections were dewaxed and rehydrated through a series of solvents, while cryosections were fixed and washed at room temperature. All sections were stained with Hematoxylin and Eosin (H&E) following standard procedures and mounted with neutral gum.

Histopathological changes was independently evaluated by three blinded investigators. Quantitative and semi-quantitative analyses were conducted using a combination of morphometric measurements and histological scoring. Placental structure was quantified by calculating the area ratios of the labyrinth and junction zones using ImageJ software. Colonic injury was assessed based on histological features, and crypt depth was quantitatively measured using ImageJ.

### Immunofluorescence analysis

The expression of colonic ZO-1 was detected by immunofluorescence staining to observe the intestinal barrier integrity. The colonic tissues were gently separated from the cecum-colon junction and immediately immersed in a 4% paraformaldehyde solution. Colonic paraffin sections were sequentially placed in dewaxing solution, anhydrous ethanol, and then rinsed with distilled water. After air-drying the sections slightly, they were incubated with BSA for 30 minutes. Anti-ZO-1 antibody (Servicebio, GB115686, diluted at 1:200) and goat anti-rabbit CY3 conjugated antibody (Servicebio, GB21303, diluted at 1:300) in PBS were used to stain colonic tissue sections, followed by restaining with DAPI (Servicebio, G1012). The sections were sealed with anti-fluorescence quenching sealing agent. Images were captured under a fluorescence microscope (NIKON ECLIPSE C1, Japan). ImageJ software was employed to quantify the fluorescence intensity of colonic ZO-1 protein.

### Intestinal permeability analysis

Intestinal permeability was evaluated using fluorescein isothiocyanate (FITC)-dextran [27]. Mice underwent fasting for 12 h and were administered FITC-dextran (50 mg/kg) via oral gavage. Blood was collected from the tail vein and centrifuged (12,000g, 5 min, 4°C). The serum FITC-dextran concentration was measured. A standard curve was generated using diluted FITC-dextran in non-treated serum diluted with PBS.

### Measurements of cytokines (IL-6, IFN-γ, TNF-α and IL-10)

Cytokine levels were measured using the LabEx Mouse Inflammatory Factor Panel LXMM10-X kit (LabEx, Shanghai, China), according to the manufacturer’s instructions. The assay plate was read using the Meso Scale Discovery (MSD) electrochemiluminescence detection system, and the data were analyzed with the MSD Discovery Workbench software (version 4.0, LabEx; Shanghai, China).

### Quantitative reverse transcription polymerase chain reaction (qPCR)

Placenta and colonic tissue were subjected to RNA isolation using TRIzol-based extraction protocols (Yisheng Biotechnology, Shanghai) in accordance with standardized operating procedures. Following RNA purification, 1 μg aliquots of DNase-treated RNA were reverse transcribed into complementary DNA using a commercial reverse transcription system (Yisheng cDNA Synthesis Kit). Quantitative amplification was performed on a Roche LightCycler 480 II thermal cycler with the following parameters: 95°C initial denaturation (10 min), 40 cycles of 95°C (15 sec) and 60°C (1 min). Transcript quantification was executed through comparative threshold cycle analysis (2^-ΔΔCt^), normalized against glyceraldehyde-3-phosphate dehydrogenase (GAPDH) as the endogenous control. Primer sequences are shown in S1 Table.

### Fecal sample collection and absolute bacterial quantification

As previously described [28], each mouse was placed in a sterilized cage on the morning. Fresh fecal pellets were promptly collected and transferred to a pre-sterilized Eppendorf tube to prevent urine contamination. The fecal samples were immediately frozen in liquid nitrogen and stored at -80°C until use.

To quantify the bacterial load in fecal matter, samples were aseptically collected from mice post-antibiotic treatment. Microbial DNA extraction was conducted utilizing the QuantiFast SYBR Green PCR Kit (Biorad) in conjunction with universal 16S rRNA primers (5′-ACTCCTACGGGAGGCAGCAG-3′ and 5′-ATTACCGCGGCTGCTGG-3′). The total bacterial count was subsequently calculated and expressed as the number of bacteria per milligram of fecal sample [29]. Plot a standard curve with the positive control’s log concentration on the x-axis and Ct value on the y-axis. Use the test sample’s Ct value on this curve to find its log DNA concentration and calculate the actual concentration.

### 16S rRNA sequencing analysis

DNA extraction from fecal samples was performed in accordance with the manufacturer’s guidelines. The extracted total DNA was eluted into 50μL of elution buffer and stored at -80°C until further PCR analysis. To prevent false-positive PCR results, ultrapure water was used as a negative control throughout the DNA extraction process. PCR products were purified using AMPure XT beads (Beckman Coulter Genomics, Danvers, MA, USA) and quantified with a Qubit fluorometer (Invitrogen, USA). Amplicon libraries for sequencing were prepared and quality-controlled as follows: Fragment size distribution was analyzed using an Agilent 2100 Bioanalyzer (Agilent Technologies, Santa Clara, CA, USA), while library quantification was performed with the Illumina-compatible Library Quantification Kit (Kapa Biosystems, Wilmington, MA, USA). High-throughput sequencing was subsequently carried out on an Illumina NovaSeq 6000 system using paired-end 250 bp (PE250) chemistry.

### LC-MS/MS analysis

An aliquot of 100 μL of serum was mixed with 400 μL of an extraction solution, which was a 1:1 (v/v) mixture of methanol and acetonitrile containing deuterated internal standards. The resulting mixture was vortexed for 30 seconds, then sonicated for 10 minutes in a 4°C water bath, and subsequently incubated at -40°C for 1 hour to precipitate proteins. After incubation, the samples were centrifuged at 12,000 rpm (RCF = 13,800 × g, R = 8.6 cm) for 15 minutes at 4°C. The supernatant was carefully transferred to a new glass vial for further analysis. For the quality control (QC) sample, equal volumes of supernatant from each sample were pooled together.

For the analysis of polar metabolites, an UHPLC system (Vanquish, Thermo Fisher Scientific) coupled with a Waters ACQUITY UPLC BEH Amide column (2.1 mm × 50 mm, 1.7 μm) and an Orbitrap Exploris 120 mass spectrometer (Thermo) was utilized. The mobile phase included 25 mmol/L ammonium acetate and 25 mmol/L ammonia hydroxide in water (pH = 9.75) as solvent A and acetonitrile as solvent B. The auto-sampler was maintained at 4°C, and the injection volume was set to 2 μL. The mass spectrometer operated in information-dependent acquisition (IDA) mode controlled by Xcalibur software (Thermo), enabling continuous evaluation of full scan MS spectra for subsequent MS/MS spectra acquisition.

The electrospray ionization (ESI) source parameters were configured as follows: sheath gas flow rate at 50 units, auxiliary gas flow rate at 15 units, capillary temperature at 320°C, full MS resolution at 60,000, MS/MS resolution at 15,000, collision energy set to 20/30/40 eV using stepped normalized collision energy (SNCE), and spray voltage adjusted to 3.8 kV in positive ion mode or -3.4 kV in negative ion mode.

### Transcriptomic RNA sequencing and analysis

Total RNA was extracted from placental tissues using TRIzol reagent (Invitrogen). RNA integrity was confirmed using an Agilent 2100 Bioanalyzer. Sequencing libraries were prepared with the VAHTS Universal V10 RNA-seq Library Prep Kit and sequenced on an Illumina NovaSeq X Plus platform (OE Biotech Co., Ltd, Shanghai), generating 150 bp paired-end reads.

Raw reads were processed with fastp to obtain clean data. Differential expression analysis was performed using DESeq2, and genes with an adjusted *p*-value (q-value) < 0.05 and |log2(FoldChange)| > 1 were considered significantly differentially expressed. Functional enrichment analysis of differentially expressed genes was conducted for GO terms and KEGG pathways.

### Evaluation of fetal and postnatal outcomes

On gestational day 18 (G18), six pregnant females were anesthetized with 2.5% Tribromoethanol, sacrificed by cervical dislocation, and fetal number and weight were recorded. In order to evaluate the offspring growth, the remaining pregnant mice (four mice per group in the UN, IN, and IN-FMT groups) were allowed to deliver naturally. Because infected dams exhibit compromised health, impaired lactation, disrupted maternal behavior, or even premature death postpartum, these factors can directly compromise offspring care and survival [30,31]. All naturally delivered newborns were transferred to healthy surrogate mothers. Newborns were monitored and body weights were recorded daily until weaning at postnatal day 20 (P20). Subsequently, five offspring from each group (the UN, IN, and IN-FMT groups) were randomly selected and euthanized, and colon lengths were measured. Another five offspring from each group were infected with PbA to observe offspring survival rates.

### Statistical analysis

Sequencing was performed on the Illumina NovaSeq platform following the manufacturer’s guidelines. Reads were allocated to their respective samples based on unique barcodes, which were then removed along with primer sequences. The paired-end reads were combined using FLASH. To ensure data quality, raw reads underwent filtering using fqtrim (version 0.94) under specific conditions to produce high-quality clean tags. Chimeric sequences were identified and removed with Vsearch software (version 2.3.4). Post dereplication via DADA2, we generated a feature table and corresponding feature sequences.

Alpha and beta diversity metrics were calculated after normalizing all samples to an equal number of sequences. Feature abundance was normalized based on relative abundance per sample, utilizing the SILVA database (release 138) classifier. Alpha diversity indices-including Chao 1, Observed species, Good’s coverage, Shannon, and Simpson-were computed using QIIME2 to assess species diversity complexity within each sample. Beta diversity calculations were also performed using QIIME2, while the visualizations were created using R packages. Sequence alignment was conducted with BLAST, and feature sequences were annotated against the SILVA database for each representative sequence. Additional graphical representations were produced using R (version 3.5.2).

The raw LC-MS/MS data were transformed into the mzXML format using ProteoWizard and subsequently processed with a custom-developed in-house program. This program, built using R and leveraging the XCMS package, facilitated peak detection, extraction, alignment, and integration. Metabolite identification was performed using an R package in conjunction with the BiotreeDB (V3.0) database.

To explore potential associations between differential bacterial genera and differential metabolites, Spearman’s rank correlation analysis was performed using R software. Correlation coefficients (r) were calculated to evaluate the strength and direction of the associations. To control for multiple hypothesis testing, *P* values were adjusted using the Holm-Šídák method, and an adjusted *P* < 0.05 was considered statistically significant. Significant correlations were visualized using a heatmap.

Quantitative date were presented as means ± standard deviation (SD). Statistical evaluations were performed using SPSS version 13.0 (IBM Corp., Armonk, NY). For comparisons among three groups, the Kruskal-Wallis test was used to assess overall group differences. Pairwise comparisons between two groups were conducted separately using the Mann-Whitney U test. The threshold for statistical significance was established at *P* < 0.05.

## Results

### *P. berghei* ANKA infection induces placental inflammation in pregnant mice

**Fig 1:**
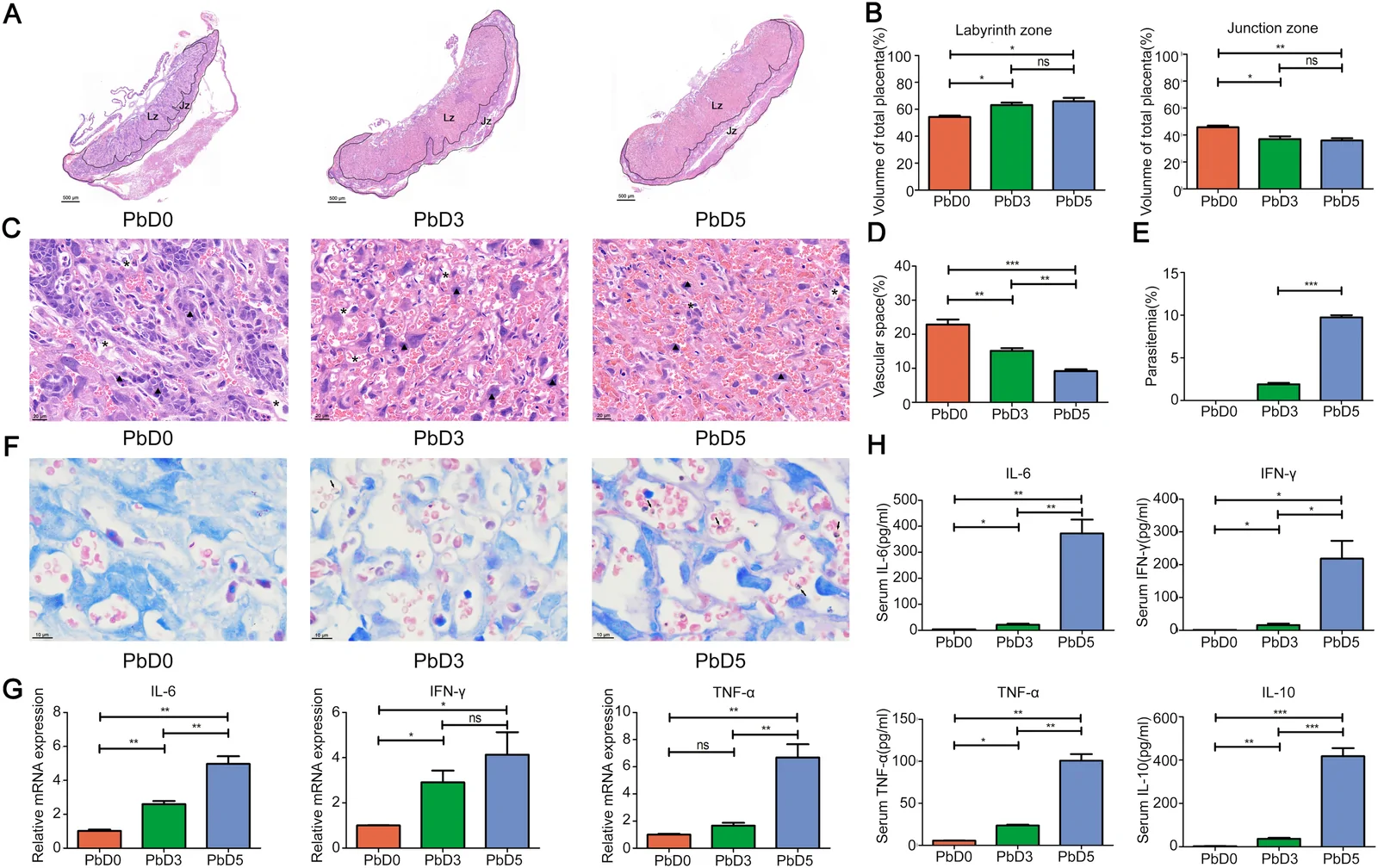
*P. berghei* ANKA infection induces placental pathology in pregnant mice. **(A)** Representative images of H&E-stained placental tissue sections. Zones are indicated by labyrinth zone (LZ) and junction zone (JZ). Scale bar: 500 µm. **(B)** Stereological measurement of the sizes of the placental zones in placentas. **(C-D)** Images illustrated the placental labyrinth region vascular spaces in PbD0, PbD3 and PbD5 groups. Scale bar: 20 µm. The asterisk (*) denotes the maternal blood sinus and a triangle (▲) denotes the trophoblast cells. **(E-F)** Images illustrated the placental labyrinth region of dams estimated placental parasitemia at PbD0, PbD3 and PbD5 groups. Scale bar: 10 µm. **(G)** The mRNA levels of placental IL-6, IFN-γ and TNF-α. **(H)** Serum levels of inflammatory factors. (n=6 per group).

After *Pb*A infection, statistically significant differences were observed in the area ratios of the placental labyrinth and junction zones compared to the PbD0 control group. Specifically, the area ratio of the placental labyrinth zone was significantly increased in the infected group, while that of the junction zone was significantly decreased. There was no statistical difference between the PbD3 and PbD5 groups (Fig 1A and 1B).

As shown in Fig 1C, with the progression of *Pb*A infection, morphological alterations were observed in the trophoblast cells, accompanied by a narrowing of the maternal blood sinuses. These changes resulted in a reduction of the space available for maternal-offspring exchange. Infected pregnant mice exhibited a reduction in placental vascular spaces at G18 compared to PbD0 controls (Fig 1C and 1D). To distinguish the effects of *Pb*A infection from normal gestational progression, we compared placental histopathology between infected and uninfected pregnant mice at G16 and G18. Relative to uninfected controls, infected mice exhibited enlarged labyrinth zones, reduced junction zones, and narrowed vascular spaces at both time points, with more pronounced alterations at G18 (S2 Fig).

The percentage of placental parasitemia in the PbD3 and PbD5 groups was 1.6% and 10.2%, respectively, indicating a significant burden of parasitic infection (Fig 1E and 1F). PbA infection significantly elevated the placental mRNA levels of IL-6, IFN-γ and TNF-α (Fig 1G), and increased serum concentrations of IL-6, IFN-γ and TNF-α and anti-inflammatory cytokine IL-10 (Fig 1H). These findings indicate activation of both pro-inflammatory and regulatory immune responses following PbA infection.

### *P. berghei* ANKA infection impairs intestinal barrier integrity and induces colonic inflammation

**Fig 2:**
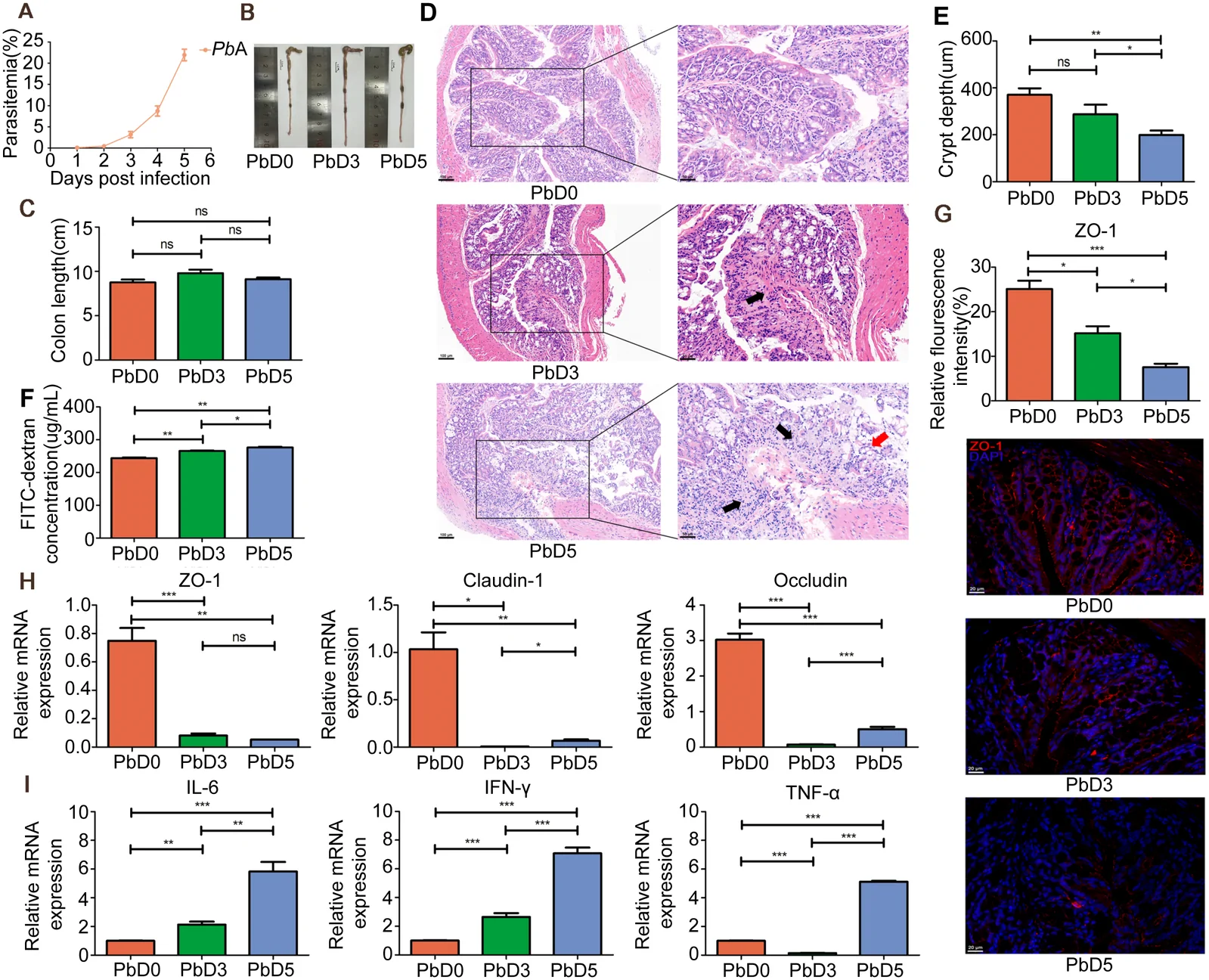
*P. berghei* ANKA infection impairs intestinal barrier integrity in pregnant mice. **(A)** Blood parasitemia. **(B)** The representative images of colon. **(C)** The colon length. **(D)** Histopathological changes of the colon tissues by H&E staining. Black arrow: inflammatory cell infiltration. Red arrow: crypt defects and mucosal ulcers. Scale bars: 100 µm and 50µm. **(E)** Colonic crypt depth. **(F)** Serum FITC-dextran concentration. **(G)** Quantification of ZO-1 fluorescence intensity and representative immunofluorescence imagesof colonic sections stained with an anti-ZO-1 antibody. Scale bar: 20 µm. **(H)** The mRNA levels of colonic ZO-1, Claudin-1 and Occludin. **(I)** The mRNA levels of colonic IL-6, IFN-γ and TNF-α. (n=6 per group).

The parasitemia of the PbD5 mice was higher than that of the PbD3 mice (Fig 2A). Compared with PbD0 controls, no significant differences were observed in the length of the colon in *Pb*A-infected groups (Fig 2B and 2C). As shown in Fig 2D, HE staining of the colon tissue revealed that the morphological structure was characterized by an intact colonic mucosa, neatly arranged colonic crypts, clear crypt outlines, and an absence of pathological alterations such as edema or ulceration in the PbD0 control group. In contrast, significant structural damage of colonic tissue was characterized by irregular crypt morphology, atrophy of crypts, disordered arrangement of goblet cells, and accompanying inflammatory cell infiltration in the infection groups. Furthermore, significant differences in colonic crypt depth were detected between the PbD5 group and the other two groups (Fig 2E).

Notably, compared to PbD0 controls, the PbD3 and PbD5 groups showed significantly higher serum levels of FITC-dextran, indicating impaired barrier integrity (Fig 2F). *Pb*A infection impaired the intact intestinal tight junctions, as evidenced by decreased ZO-1 protein expression (Fig 2G), and reduced mRNA levels of ZO-1, Claudin-1 and Occludin in the colon (Fig 2H). Furthermore, the PbD5 group displayed obvious colonic inflammation, characterized by upregulated mRNA expression levels of IL-6, IFN-γ and TNF-α (Fig 2I). Taken together, these results suggest that *Pb*A infection disrupts the intestinal barrier.

### *P. berghei* ANKA infection alters gut microbiota community in pregnant mice

**Fig 3:**
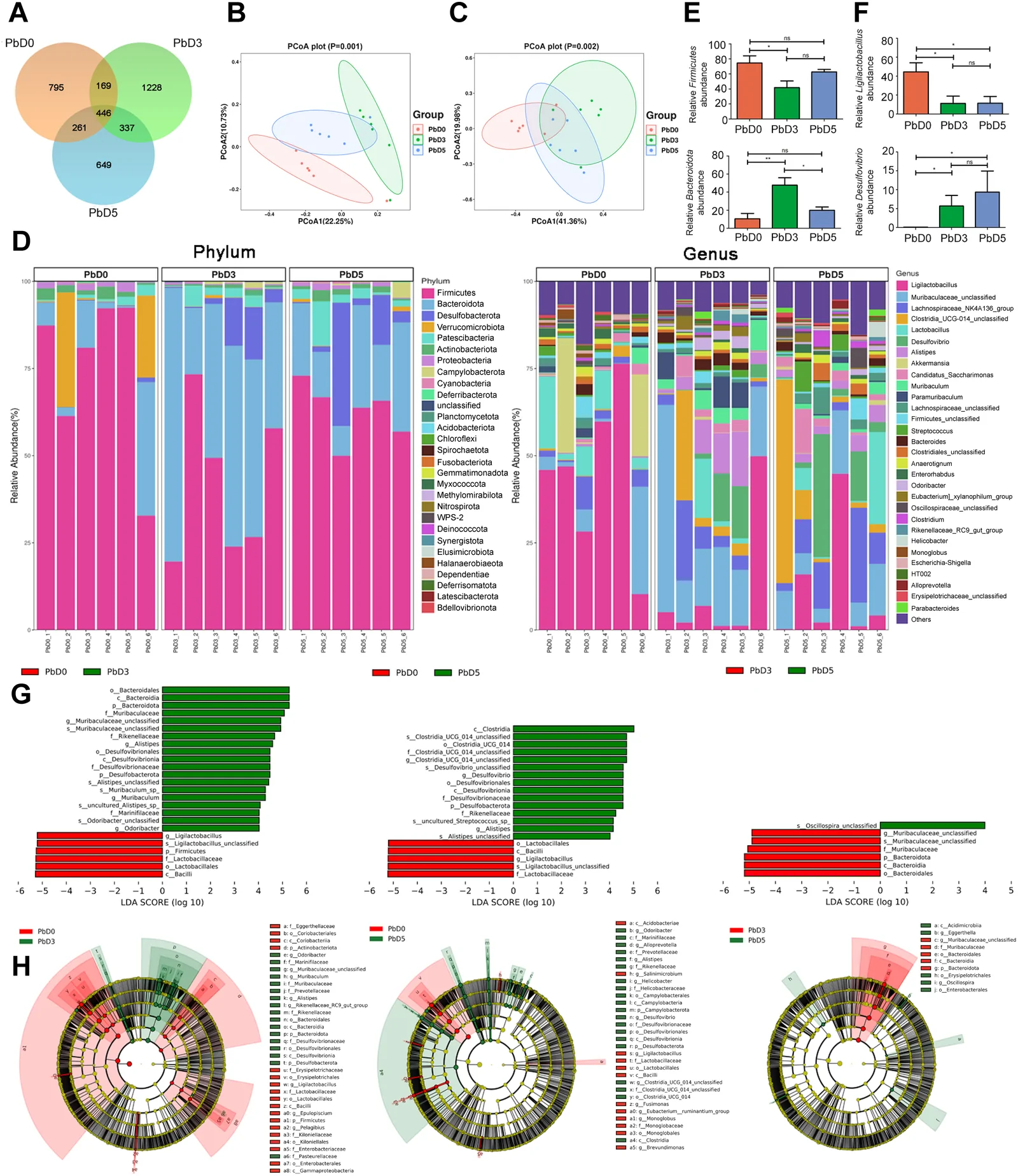
*P. berghei* ANKA infection induces gut microbiota dysbiosis in pregnant mice. **(A)** Venn diagram showing the unique and shared ASVs among the experimental groups. Principal coordinates analysis (PCoA) based on unweighted **(B)** and weighted **(C)** UniFrac distance matrixes of fecal samples. **(D)** Variations in the gut microbiota composition at the phylum and genus levels. **(E)** Relative abundance of *Firmicutes* and *Bacteroidota* at the phylum level. **(F)** Relative abundance of *Ligilactobacillus* and *Desulfovibrio* at the genus level. **(G)** LDA score distribution. **(H)** LEfSe cladogram showing differentially abundant bacterial taxa. (n=6 per group).

Next, the composition of fecal microbiota after *Pb*A infection was analyzed using 16S rRNA sequencing. A Venn diagram illustrates the similarities and differences in microbial communities among the three groups (Fig 3A). A total of 3090 amplicon sequence variants (ASVs) were detected in the PbD3 and PbD5 groups, with 1,228 ASVs unique to the PbD3 mice and 649 ASVs unique to the PbD5 mice. Alpha diversity metrics, including chao1, Good’scoverage, ObservedOTUs, Shannon and Simpson diversity indices, and phylogenetic diversity values (S2 Table), revealed that *Pb*A infection changed the richness and evenness among the PbD0, PbD3 and PbD5 groups. The similarity of microbial composition of each sample was assessed by the PCoA based on unweighted and weighted UniFrac distance matrices (Fig 3B and 3C). There were distinct differences in microbial community composition among three groups at the phylum and genus levels (Fig 3D). At the phylum level, compared with the PbD0 controls, the relative abundance of Firmicutes was significantly decreased in the PbD3 group, while Bacteroidetes was notably increased in the PbD5 group (Fig 3E). At the genus level, the gut microbiota of infected mice was characterized by increased relative abundances of *Desulfovibrio, Lachnospiraceae_NK4A136_group and Clostridia_UCG-014_*unclassified, and decreased *Ligilactobacillus, Lactobacillus and Akkermansia* (Fig 3D and 3F).

To exclude the potential effect of pregnancy on gut microbiota composition, we additionally analyzed infected non-pregnant mice at the corresponding infection time points (non-pregnant PbD3 and non-pregnant PbD5). Compared with infected non-pregnant mice, pregnant infected mice exhibited no significant differences in the relative abundance of the major bacterial taxa at either the phylum or genus level (S3 Fig). Specifically, neither *Firmicutes* nor *Bacteroidota* at the phylum level, nor representative genera including *Ligilactobacillus, Lactobacillus, Akkermansia,* and *Desulfovibrio*, differed significantly between the corresponding pregnant and non-pregnant infected groups (all *P* > 0.05). Collectively, these results suggest that pregnancy status did not substantially influence the core microbiota alterations associated with PbA infection.

### Differentially abundant microbial genera identified by LEfSe analysis

LEfSe analysis identified statistically significant microbial biomarkers at different taxonomic levels among the three groups (Fig 3G and 3H). At the family level, *Muribaculaceae* (*Bacteroidota* phylum) was identified as a significantly different biomarker between the PbD3 and PbD0 groups; *Clostridia*_*UCG-014*_unclassified (belonging to the Firmicutes phylum) showed a significant difference between the PbD5 and PbD0 groups; *Oscillospira*_unclassified (belonging to the *Firmicutes* phylum) represented a distinctive microbial biomarker distinguishing the PbD5 and PbD3 groups.

### Untargeted metabolomics reveals dynamic alterations in the serum metabolome of *Pb*A-infected pregnant mice

**Fig 4:**
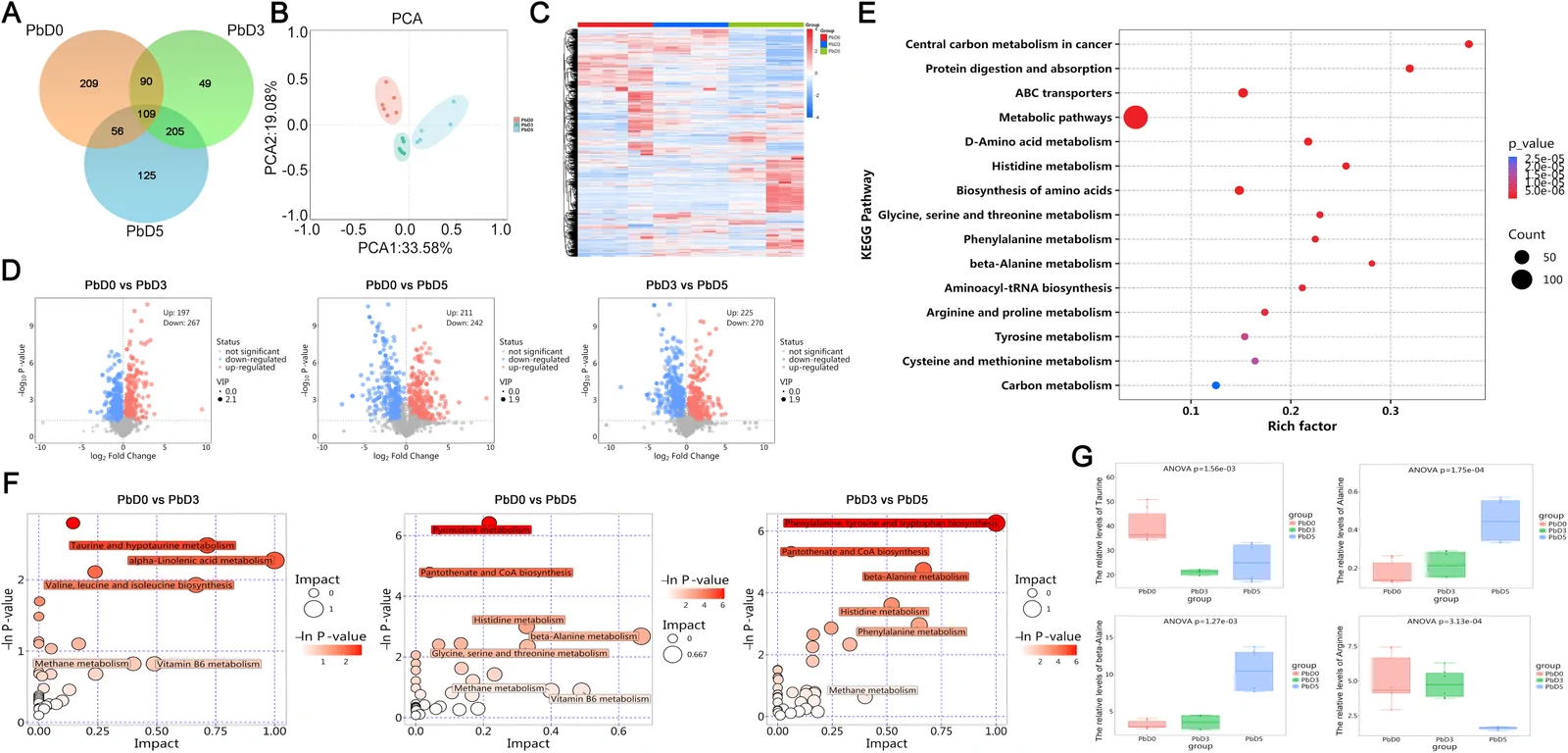
*P. berghei* ANKA infection induces alterations in the serum metabolome in pregnant mice. Venn diagram showing shared and unique serum metabolites among the experimental groups. **(B)** PCA score scatter plots. **(C)** Heatmap of differentially abundant serum metabolites. (D) Volcano plot of differential metabolites. Red represents upregulated, and blue represents downregulated. Each point represents a metabolite. **(E)** KEGG pathway enrichment analysis. The size of the dots represents the number of metabolites. RichFactor represents the ratio of differential metabolites to total metabolites within each pathway. **(F)** Bubble plot presenting significantly enriched metabolic pathways between two groups. The size and color indicate pathway impact and *P* value, respectively. **(G)** Relative abundance of four representative differential metabolites identified by untargeted metabolomics. The Y-axis represents the relative abundance of the corresponding metabolites across groups.(n=6 per group).

To comprehensively characterize the metabolic alterations during *Pb*A infection, untargeted metabolomic profiling was performed on serum samples collected from pregnant mice. Among the 843 detected metabolites, 109 were shared across all three groups, indicating distinct metabolic profiles among the experimental groups (Fig 4A). Unsupervised hierarchical clustering combined with PCA showed clear separation of the three groups (Fig 4B). Considering the concurrent effects of infection progression and advancing gestation, the observed metabolic alterations should be interpreted as reflecting the combined influence of both factors rather than being attributed solely to *Pb*A infection. In addition, hierarchical clustering and volcano plot analyses further demonstrated distinct metabolic profiles among the three groups (Fig 4C and 4D).

KEGG pathway enrichment analysis identified the top 15 significantly altered metabolic pathways (Fig 4E). At PbD3, taurine and hypotaurine metabolism, alpha-linolenic acid metabolism, and valine, leucine, and isoleucine biosynthesis were the most prominently enriched pathways (Fig 4F). At PbD5, pyrimidine metabolism, pantothenate and CoA biosynthesis, and histidine metabolism were the most prominently enriched pathways. Several of these altered pathways have previously been implicated in intestinal and placental barrier dysfunction [32–34]. Given the prominent enrichment of amino acid metabolism, we focused on the top 4 key differential metabolites (Taurine, alanine, β-alanine and arginine) for further investigation (Fig 4G).

### Correlation analysis between bacterial genera and differential metabolites

**Fig 5:**
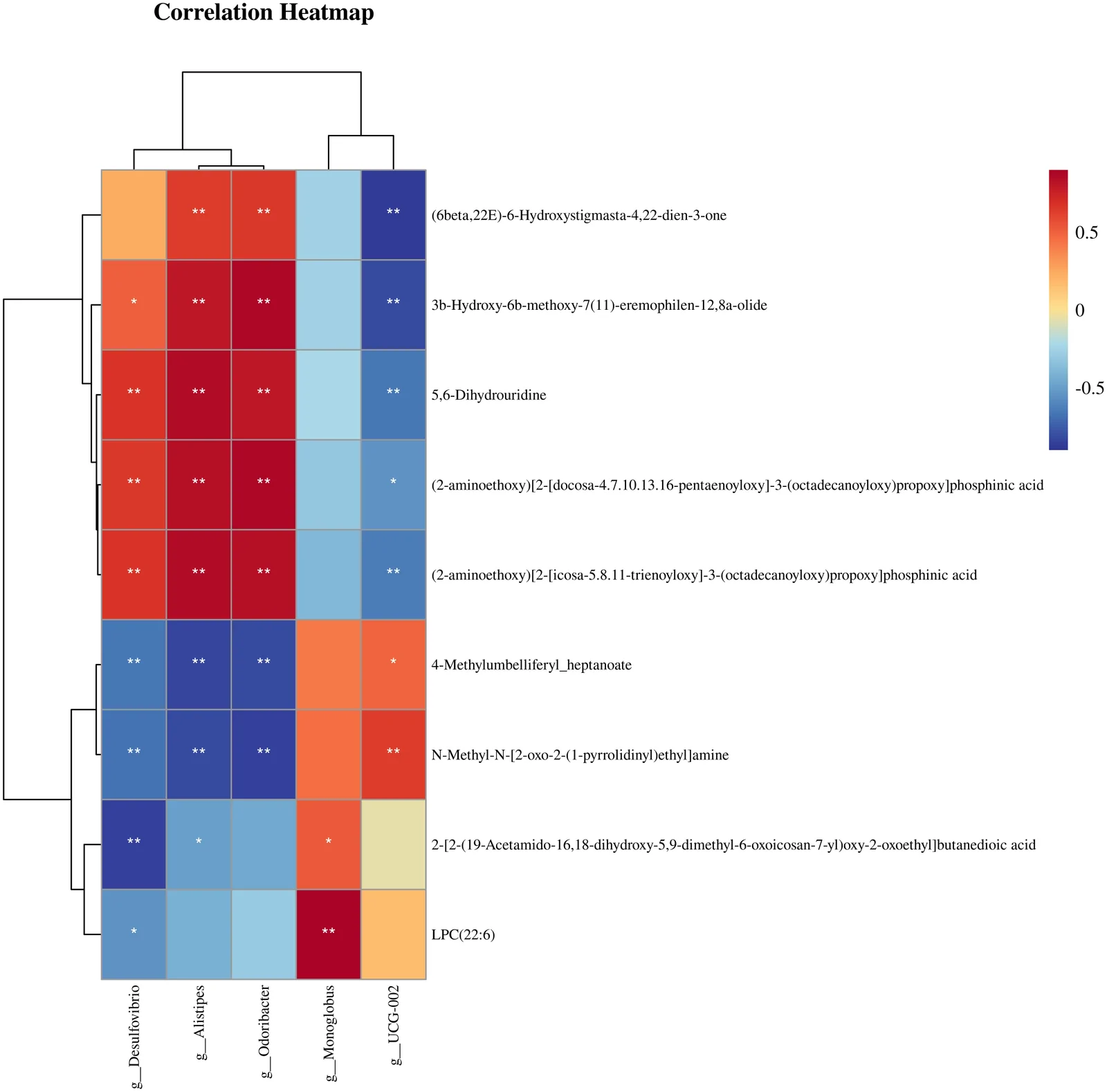
Correlation heatmap of differential metabolites and gut bacterial genera. The correlations are exhibited by colors; blue indicates positive correlations, red indicates negative correlations, and color intensity illustrates correlation strength. *P < 0.05.

To explore the associations between differential bacterial genera and metabolites, Spearman’s correlation analysis was performed and visualized as a heatmap (Fig 5). Notably, the relative abundance of *Monoglobus* was positively correlated with LPC(22:6) (r = 0.89, adjusted *P* < 0.05), suggesting a potential role for this genus in maintaining lipid metabolic homeostasis during infection. In contrast, the potentially pathogenic genus *Desulfovibrio* exhibited a strong negative correlation with 2-(2-(19-Acetamido-16,18-dihydroxy-5,9-dimethyl-6-oxoicosan-7-yl) oxy-2-oxoethyl) butanedioic acid (r = -0.86, adjusted *P* < 0.05), implying that the expansion of *Desulfovibrio* may contribute to the depletion of this metabolite during disease progression. In addition, *Alistipes* and *Odoribacter* respectively exhibited significant positive and negative correlations with multiple metabolites involved in lipid and nucleoside metabolism (|r| = 0.84-0.87, adjusted *P* < 0.05). Collectively, these results revealed significant associations between differential gut bacterial genera and host metabolites following *Pb*A infection, highlighting coordinated alterations in the gut microbiota and metabolome.

### Gut microbiota mediates colonic injury in *Pb*A-infected pregnant mice

**Fig 6:**
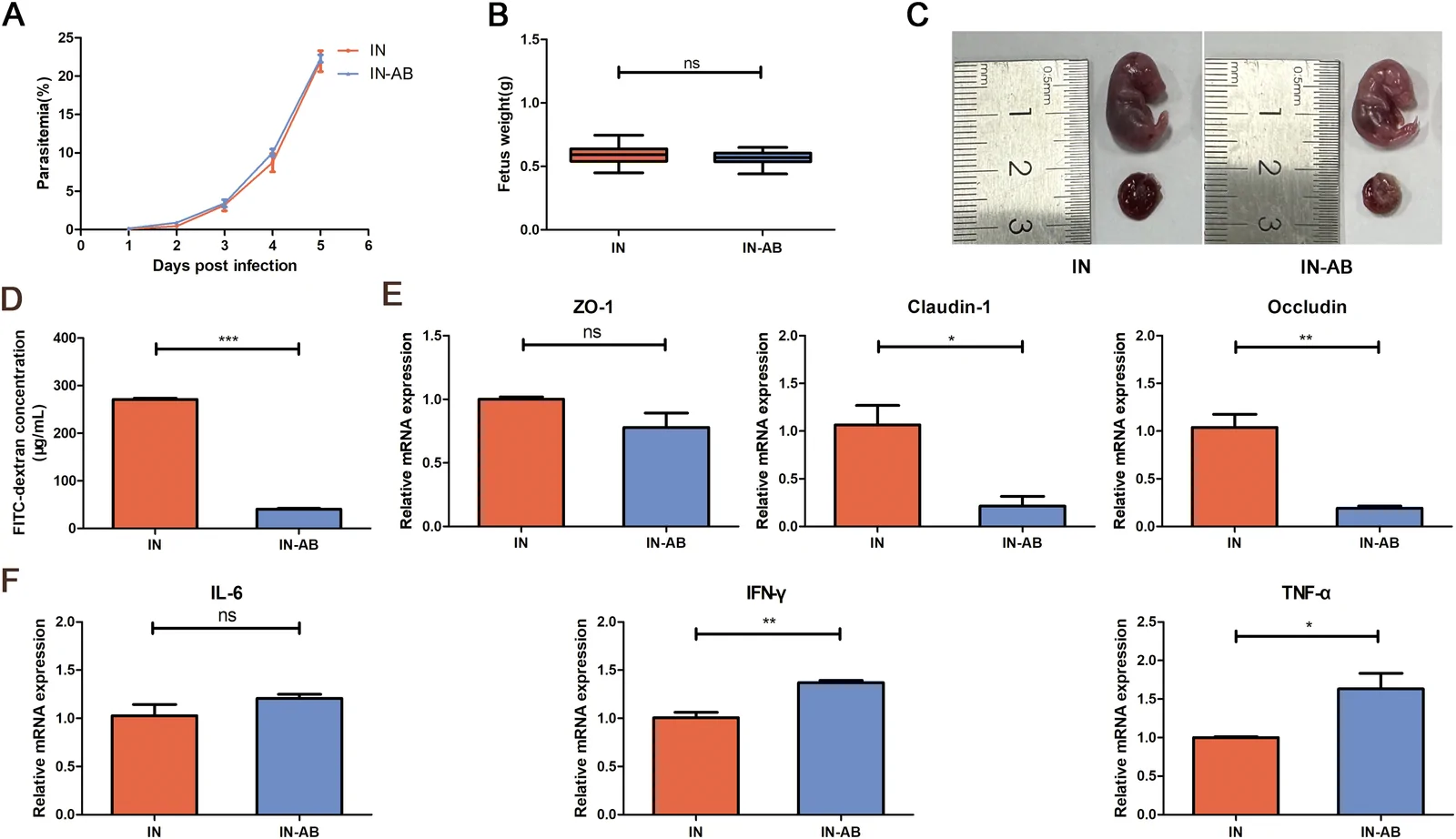
Gut microbiota depletion exacerbates *Pb*A infection-induced intestinal injury and inflammation in pregnant mice. **(A)** Blood parasitemia. **(B)** Fetal weight. **(C)** The representative images of fetuses. **(D)** Serum FITC-dextran concentration. **(E)** The mRNA levels of colonic ZO-1, Claudin-1 and Occludin. **(F)** The mRNA levels of colonic IL-6, IFN-γ and TNF-α. (n = 6 per group).

To assess the impact of gut microbiota depletion on host immunity, intestinal barrier function, and fetal health, *Pb*A-infected pregnant mice were treated with broad-spectrum antibiotics. Antibiotic treatment reduced the bacterial load by approximately 30-fold, confirming effective gut microbiota depletion (S4 Fig). No significant differences in parasitemia or pregnancy outcomes (fetal weights) were observed between the IN and IN-AB groups (Fig 6A-C). However, the IN-AB group exhibited higher serum FITC-dextran levels (Fig 6D), significant downregulation of colonic tight junction proteins (Claudin-1 and Occludin; Fig 6E), and elevated IFN-γ and TNF-α mRNA levels (Fig 6F). Collectively, these results indicate that depletion of the gut microbiota did not affect parasitemia or pregnancy outcomes, but was associated with aggravated intestinal barrier dysfunction and colonic inflammation during *Pb*A infection.

### Fecal microbiota transplantation ameliorates intestinal barrier integrity impairment and colonic inflammation induced by *P. berghei* ANKA infection

**Fig 7:**
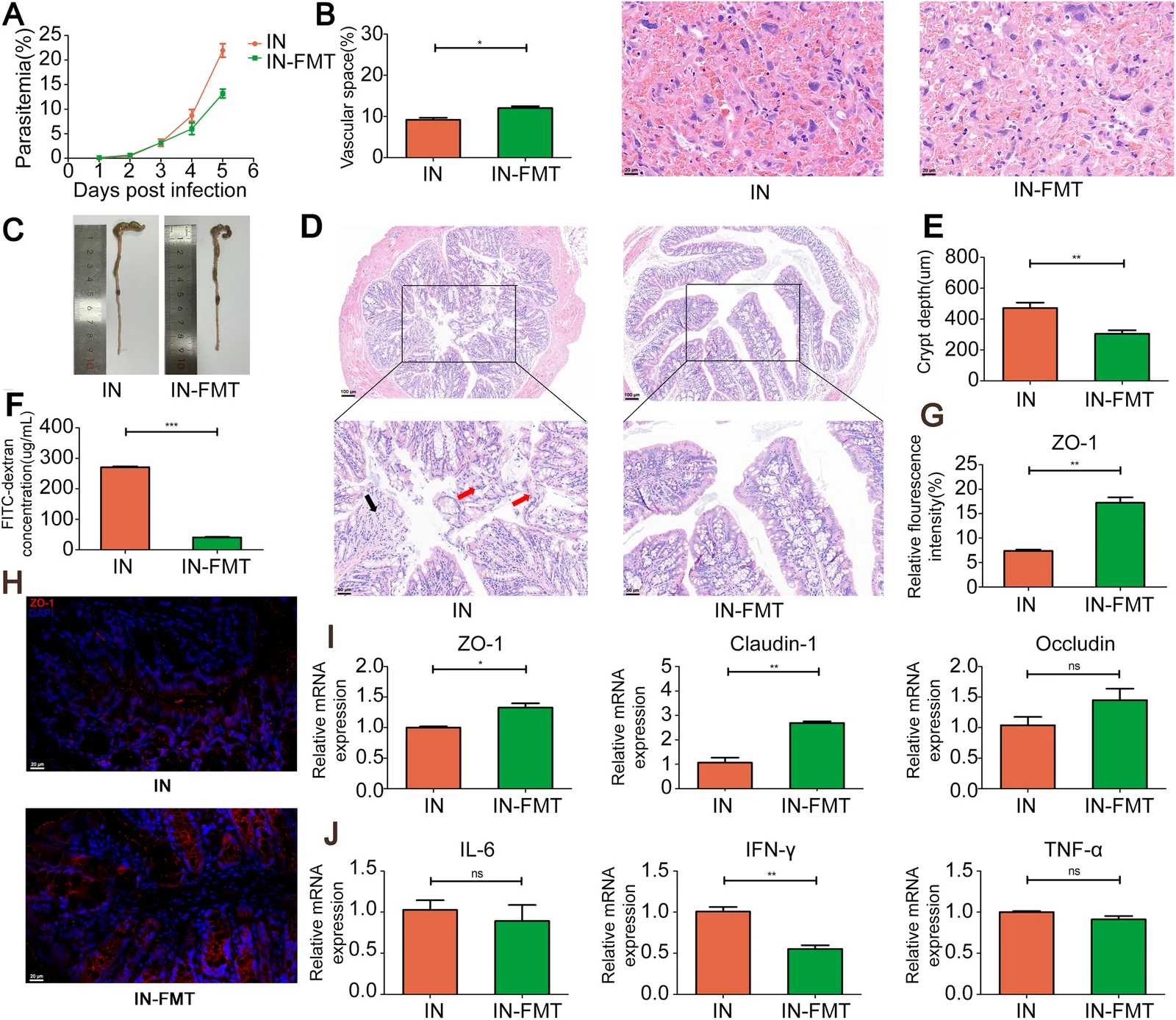
Fecal microbiota transplantation alleviates *Pb*A infection-induced intestinal barrier dysfunction in pregnant mice. **(A)** Blood parasitemia. **(B)** placental labyrinth region vascular spaces. Scale bar: 20 μm. **(C)** The representative images of colons. **(D)** Histopathological changes of the colon tissues by H&E staining. Black arrow: inflammatory cell infiltration. Red arrow: crypt defects and mucosal ulcers. Scale bars: 100 μm and 50 μm. **(E)** Colonic crypt depth. **(F)** Serum FITC-dextran concentration. **(G-H)** Quantification of ZO-1 fluorescence intensity and representative immunofluorescence images of colonic sections stained with an anti-ZO-1 antibody. Scale bar: 20 μm. **(I)** The mRNA levels of colonic ZO-1, Claudin-1 and Occludin. **(J)** The mRNA levels of colonic IL-6, IFN-γand TNF-α. (n = 10 per group).

To investigate whether restoring microbial homeostasis could mitigate infection-induced pathology, FMT from healthy donors ameliorated *Pb*A-induced colonic pathology in pregnant mice. The IN-FMT group showed decreased parasitemia and significantly increased placental vascular spaces (Fig 7A and 7B). There was no significant difference in the length of the colon between the two groups (Fig 7C). However, compared with the IN group, the IN-FMT group exhibited significant structural recovery of the colonic mucosa, including orderly and denser arrangement of epithelial cells, regular morphology of intestinal crypts, along with a reduction in inflammatory cell infiltration and increased crypt depth (Fig 7D and 7E). Compared to IN group, the IN-FMT group showed significantly lower serum levels of FITC-dextran (Fig 7F). FMT intervention attenuated the infection-induced impairment of intestinal tight junctions, as evidenced by the elevated expression of ZO-1 protein (Fig 7G and 7H) and upregulation of ZO-1, Claudin-1, and Occludin mRNA levels (Fig 7I). Furthermore, FMT also reduced the mRNA expression level of pro-inflammatory cytokine IFN-γ in colonic tissue (Fig 7J).

### Maternal *Pb*A infection affects offspring weight and susceptibility to *Pb*A infection

**Fig 8:**
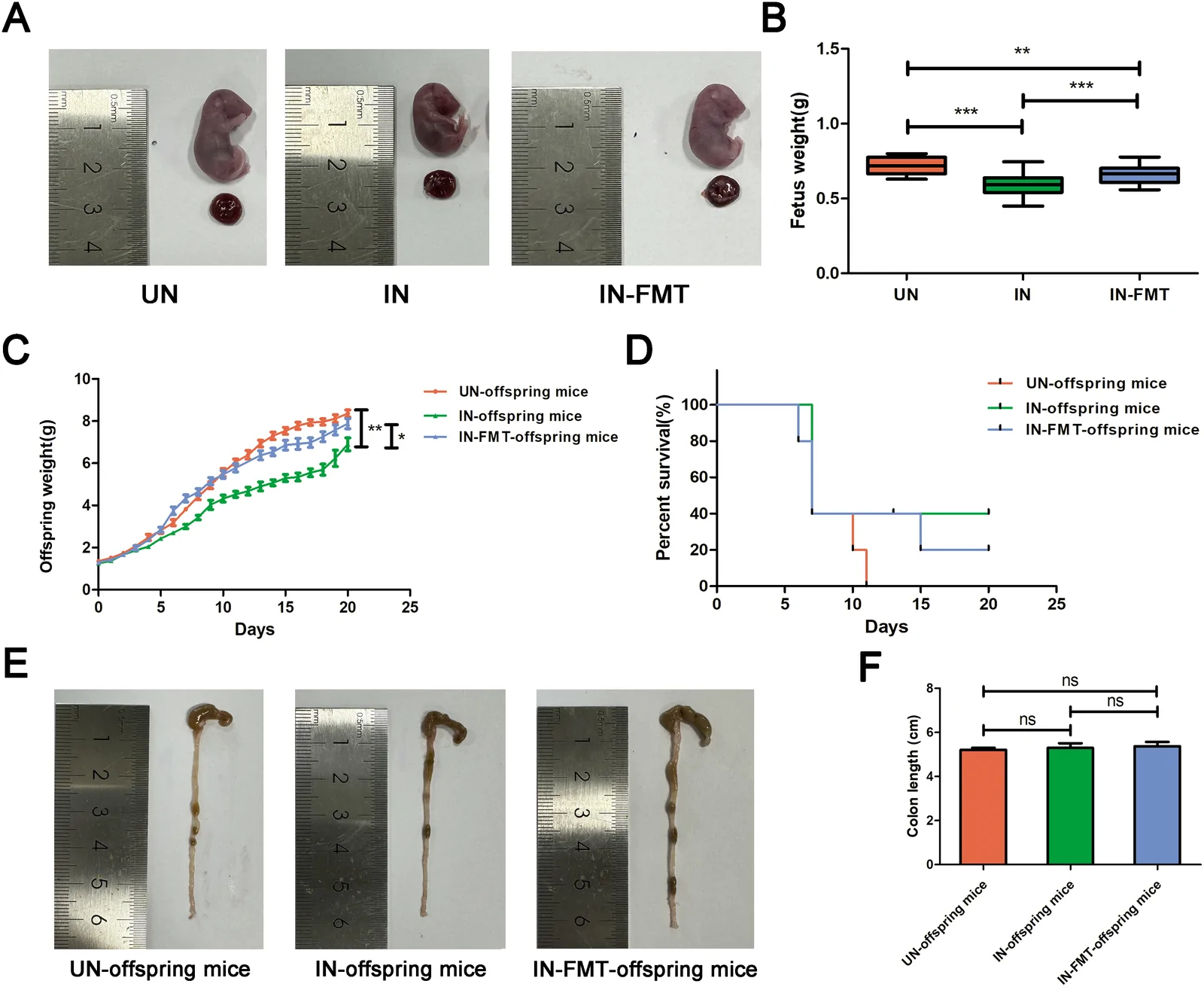
Effects of maternal *P. berghei* ANKA infection on offspring development and susceptibility to *Pb*A infection. **(A)** The representative images of fetuses. **(B)** Fetal weight. **(C)** Body weight of offspring from birth to postnatal day 20. Data are presented as mean ± SD (n = 10 per group at each time point). **(D)** The survival rate of offspring following *Pb*A ANKA infection. **(E)** The representative images of colons. **(F)** The colon length. (n = 5 per group).

To evaluate the impact of maternal infection on late gestation and postnatal development, we conducted assessments of fetal outcomes at G18 (late gestation) and postnatal growth/survival in naturally delivered offspring. At G18, fetal weight was significantly decreased in the IN group compared with the UN group. Notably, this infection-induced fetal growth restriction was partially rescued by FMT intervention (Fig 8A and 8B). The number of fetuses per dam (litter size) showed no significant difference across experimental groups (approximately 6 fetuses per litter; S5 Fig), which minimizes potential confounding effects of variable litter sizes on fetal weight comparisons.

To further assess the long-term impact of maternal infection on offspring, postnatal growth and susceptibility to *Pb*A infection were evaluated. Body weight was monitored in naturally delivered offspring from birth until weaning (postnatal day 20). The results showed that offspring from the IN group exhibited significantly lower postnatal weight compared to the other two groups (Fig 8C). After weaning at postnatal day 20, offspring were infected with *Pb*A, and their survival was subsequently monitored (Fig 8D). Compared with UN groups, offspring from the IN and IN-FMT groups exhibited increased survival following *Pb*A infection. No significant differences in colon length were observed among the three groups(Fig 8E and 8F).

**Fig 9:**
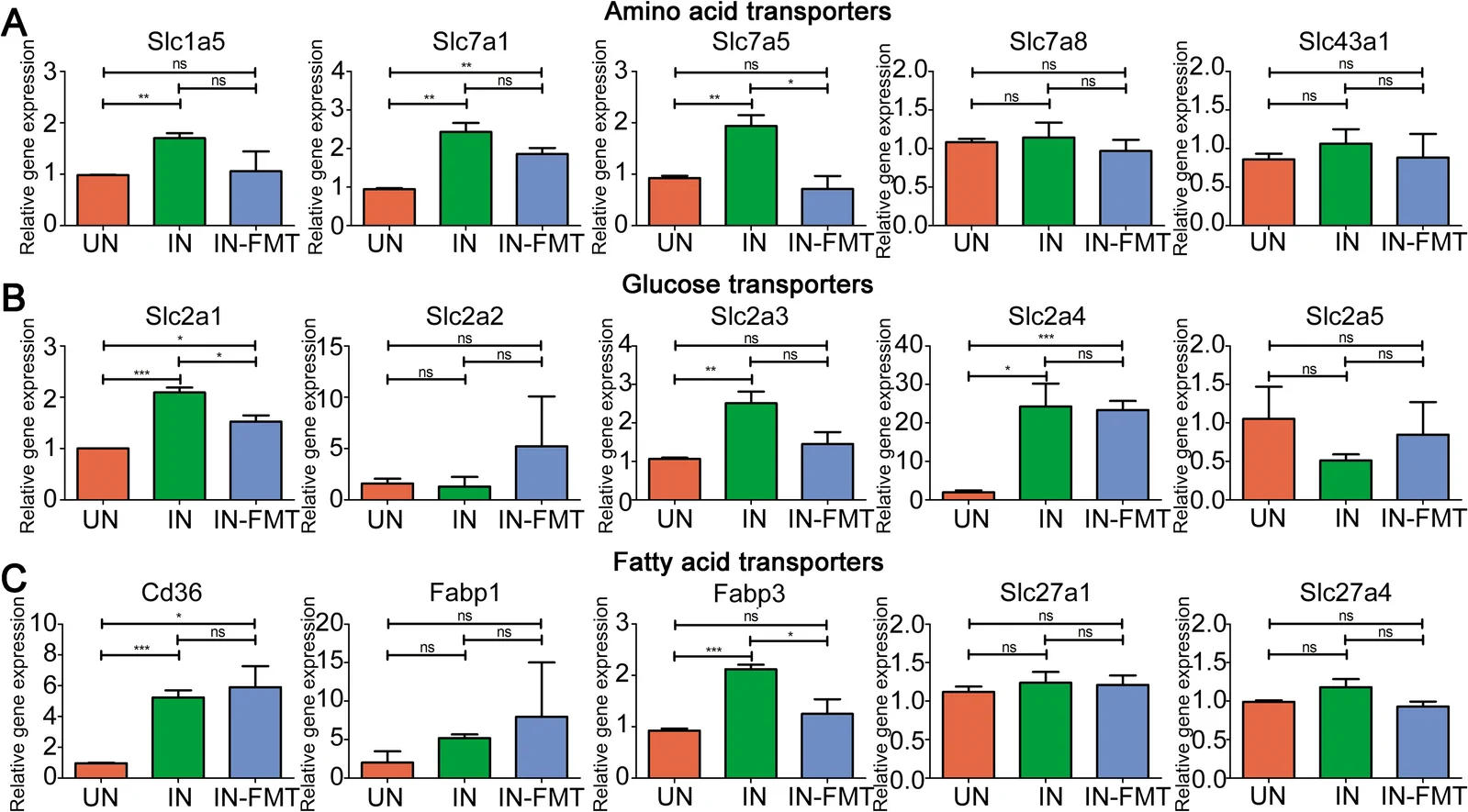
Transcription levels of placental nutrient transport genes. **(A)** The mRNA expression levels of amino acid transport genes. **(B)** The mRNA expression levels of glucose transport genes. **(C)** The mRNA expression levels of fatty acid transport genes. (*P < 0.05, **P < 0.01,***P < 0.001, ns: no significance) (n = 6 per group).

To investigate the molecular mechanisms underlying the reduced postnatal weight, we performed transcriptomic sequencing of placental tissues with a focus on nutrient transport pathways. Transcriptome analysis revealed extensive alterations in genes associated with placental nutrient transport. Specifically, compared with the UN and IN-FMT groups, the expression of the amino acid transport *Slc7a5*, glucose transport *Slc2a1*, and fatty acid transport *Fabp3* was significantly upregulated in the IN group (Fig 9), suggesting dysregulation of placental nutrient transport in response to maternal *Pb*A infection.

## Discussion

In the present study, we demonstrate that *Pb*A infection disrupts the gut-placenta axis in pregnant mice, leading to a spectrum of adverse outcomes. Compared to uninfected controls, infected dams developed significant parasitemia, reduced placental vascular spaces, and decreased fetal weight. Although FMT partially rescued offspring weight, placental inflammatory responses persisted, suggesting only partial restoration of placental function. *Pb*A infection also induced marked intestinal histopathological injury, accompanied by reduced expression of key tight junction proteins. Furthermore, depletion of the gut microbiota by broad-spectrum antibiotics exacerbated *Pb*A infection-induced intestinal injury, whereas FMT from healthy donors partially restored barrier integrity and attenuated intestinal inflammation. Collectively, these findings support an important role for the gut microbiota in maintaining intestinal barrier integrity and modulating intestinal inflammatory responses during *Pb*A infection, consistent with previous studies [35,36].

Furthermore, 16S rRNA sequencing revealed that PbA infection disrupted gut microbial homeostasis, characterized by a reduced abundance of *Firmicutes* and an increased abundance of *Bacteroidetes*, depletion of the beneficial genus *Ligilactobacillus,* and enrichment of the potentially pathogenic genus *Desulfovibrio.* Correlation analysis further demonstrated significant associations between altered bacterial genera and host metabolites involved in lipid and amino acid metabolism, highlighting coordinated changes in the gut microbiota and metabolome during *Pb*A infection.

Among these altered taxa, *Ligilactobacillus* and *Desulfovibrio* are of particular biological interest because of their established roles in maintaining intestinal homeostasis and regulating inflammatory responses. As a key probiotic, *Ligilactobacillus* contributes to intestinal homeostasis by enhancing the barrier function and suppressing inflammation. The depletion of *Ligilactobacillus* may exacerbates endotoxemia-induced inflammation by weakening mucosal defenses [31]. The loss of *Ligilactobacillus* may disrupt microbial collaboration in the gut, thereby potentially affecting microbial tryptophan metabolism and the production of indole-3-propionic acid (IPA) [37,38], while also contributing to alterations in microbial taurine metabolism and related metabolite profiles [39,40].

Conversely, the expansion of *Desulfovibrio* was associated with reduced levels of a lipid-related metabolite, suggesting disrupted lipid metabolism and inflammatory regulation during *Pb*A infection. In addition, hydrogen sulfide produced by Desulfovibrio can directly impair intestinal epithelial integrity and promote inflammatory responses [41,42]. Together, these observations support an association between Desulfovibrio expansion, metabolic dysregulation, and impaired gut ecosystem stability [43,44].

Collectively, these microbial and metabolic disturbances may contribute to intestinal barrier dysfunction and dysregulated colonic immune responses during *Pb*A infection, accompanied by increased expression of pro-inflammatory cytokines and elevated IL-10 levels [2]. The restoration of beneficial microbial taxa through FMT partially mitigated infection-induced immune dysregulation during pregnancy, alleviated colonic inflammation, and partially restored barrier function.

These findings provide mechanistic insight into the role of the gut-placenta axis in malaria during pregnancy and highlight the potential of FMT. Nevertheless, several limitations should be considered. Firstly, owing to the complexity of the malaria-infected pregnancy model, including infection-associated maternal mortality and reduced reproductive success, the number of dams available for offspring-related analyses was limited. In addition, the observed microbial and metabolic alterations may be affected by infection progression and gestational advancement. Future studies incorporating larger cohorts and gestational age-matched controls are required to further distinguish the effects of infection from those of pregnancy. Secondly, the controlled experimental conditions may not fully reflect the complex environmental and immunological factors in malaria-endemic populations. Therefore, the translational relevance of these findings to human malaria in pregnancy remains uncertain, and further validation in human cohorts and clinical studies is warranted. Thirdly, gut microbiota profiles following FMT were not systematically characterized. Future studies are required to determine the impact of FMT on the gut microbiota.

To address these limitations and enhance clinical translation, future studies should prioritize well-designed clinical trials, with particular attention to: (1) stringent participant selection criteria, (2) optimized treatment protocols, and (3) comprehensive outcome measures, including maternal anemia, offspring growth parameters, and longitudinal microbiome profiling. Furthermore, exploring alternative microbiome-modulating strategies, such as targeted probiotics, prebiotics, or microbiota-derived metabolites, may offer more practical and clinically translatable therapeutic approaches.

## Conclusion

Our study demonstrates that *Pb*A infection disrupts the gut-placenta axis in pregnant mice, resulting in intestinal barrier dysfunction, gut microbiota dysbiosis, systemic metabolic alterations, placental inflammation, and adverse pregnancy outcomes. Importantly, restoration of the gut microbiota by FMT partially alleviated intestinal and placental pathology and improved pregnancy outcomes. These findings provide new mechanistic insights into the contribution of the gut-placenta axis to malaria pathogenesis during pregnancy, and support the potential of microbiota-targeted interventions as experimental therapeutic strategies. Future research should validate these findings in clinical settings and explore additional strategies to modulate the gut microbiome, thereby optimizing therapeutic interventions.

## Supporting information

S1 Table The qPCR primer sequences involved in this study. (XLSX)

S2 Table Amplicon Sequence Variant (ASV)-base diversity indices in PbD0, PbD3 and PbD5 fecal samples. (XLSX)

S1 Fig Experimental design and timeline of antibiotic and fecal microbiota transplantation (FMT) interventions in *Pb*A-infected pregnant mice. Uninfected (UN) controls received PBS at gestational time points matched to infected groups. *Pb*A-infected mice were inoculated on gestation day 13 (G13) and sampled at G13 (PbD0 group), G16 (PbD3 group), or G18 (PbD5 group). For microbiota depletion, a separate cohort of mice was treated with an antibiotic cocktail (100 mg/kg vancomycin, 200 mg/kg neomycin sulfate, 200 mg/kg metronidazole, and 200 mg/kg ampicillin) via oral gavage for 3 days. FMT was performed daily from G13 to G18.
(TIF)

S2 Fig Stereological analysis of placental labyrinth and junction zone volumes in *Pb*A-infected and uninfected mice at G16 and G18. Scale bar: 20 μm. The asterisk (*) denotes the maternal blood sinus and a triangle (▲) denotes the trophoblast cells.
(TIF)

S3 Fig Comparative analysis of gut microbiota at the phylum and genus levels between pregnant and non-pregnant mice with *Pb*A infection. (TIF)

S4 Fig Quantification of fecal bacterial load following antibiotic-induced microbiota depletion. Data are presented as mean±SEM; statistical significance was assessed by one-way ANOVA (***p < 0.001).
(TIF)

S5 Fig The number of fetal mice across experimental groups. (TIF)
