# Supplementary material for: Fecal microbiota transplantation alleviates adverse pregnancy outcomes and intestinal injury in experimental malaria
Source: PLoS Pathog. 2026 Aug 20;22(8):e1014459. doi: 10.1371/journal.ppat.1014459 (PMC13492792; doi:10.1371/journal.ppat.1014459)
Supplement: Supplementary file 1 [file ppat.1014459.s009.docx]

Dear Dr.

Thank you for giving us the opportunity to submit a revised version of our manuscript, “Gut Microbiota Modulation in Malaria-Infected Pregnant mice: Implications for Maternal and Neonatal Health” (PPATHOGENS-D-25-00948), for consideration for publication in PLOS Pathogens. We also wish to extend our sincere gratitude to the reviewers for their time and insightful comments, which have been invaluable in helping us strengthen our work.

We have now carefully addressed all the points raised. Below, you will find our point-by-point responses to the reviewers’ comments. All corresponding changes have been incorporated into the manuscript, and the revised text has been highlighted for easy reference.

We wish to note that due to the large file sizes of Figures 1 and 4 (containing HE staining images and metabolomics heatmaps respectively), they have been compressed and uploaded together as a single supplementary file to meet the submission system requirements. We have also addressed the reviewer's concern regarding image resolution by providing high-resolution versions of all figures in the revised submission.

Jiangnan University Medical Center, Department of Public Health and Preventive Medicine, Wuxi School of Medicine, Jiangnan University, Wuxi, China

E-mail: woerseng@126.com

Thank you very much for your attention and consideration.

Best regards.

Yours sincerely,

Cheng yang

Reviewers' Comments:

Reviewer's Responses to Questions

Part I - Summary

Reviewer #1: The article by Han S, et al., reports on the effect of P. berghei ANKA-induced changes on gut microbiota and the effect these have on pregnancy malaria outcomes. Specifically, the authors demonstrate that P. berghei ANKA causes changes in the gut microbiome that associates with changes in serum metabolites. Treatment of pregnant mice infected with P. berghei with antibiotics had no effect on fetal weight but decreased expression of claudin-1 and occluding with concomitant increase in the expression of IFN-g and TNF. They also report that fecal microbiota transplants from uninfected pregnant mice decreased P. berghei-induced intestinal damage and resulted in increased fetal weight compared to untreated P. berghei-infected pregnant mice. A major concern is the lack of mechanistic insight provided in this report especially as a prior report has described gut microbiota contributions towards pregnancy malaria outcomes (PMID: 31160271).

Response: Thank you for your thoughtful review and for highlighting the important prior research (PMID: 31160271). The cited work established that gut microbiota composition affects malaria susceptibility in pregnancy, and that modulation of the microbiota can influence infection severity and pregnancy outcomes. However, the gut microbiota influences fetal development, intestinal integrity, and host metabolism via the gut-placenta axis remain incompletely elucidated.

Our integrated analysis reveals that malaria-induced dysbiosis drives pathology through the gut-placenta axis:

1.Barrier Disruption: malaria-induced dysbiosis disrupts intestinal barrier function and intestinal barrier integrity (evidenced by reduced claudin-1/occludin) and increases pro-inflammatory cytokines (IFN-γ/TNF-α).

2.Metabolic Reprogramming: Serum metabolomics revealed significantly reduced levels of nutritional substrates such as amino acids and fatty acids. The dysbiosis triggers a pro-inflammatory metabolic shift, characterized by the depletion of key protective metabolites, including indole-3-propionic acid (IPA) and taurine, which are crucial for maintaining mucosal integrity and reducing inflammation.

3.Placental Stress: The mRNA expression of placental nutrient transporters—specifically the amino acid transporter Slc7a5, glucose transporter Slc2a1, and fatty acid transporter Fabp3—was upregulated. Concurrently, serum nutrient levels were reduced, suggesting a state of nutrient stress which may contribute to fetal growth restriction. These observations suggest that under conditions of insufficient maternal nutrient supply, the placenta may upregulate transporter expression to sustain nutrient acquisition.

Critically, we further evaluate the survival analysis of offspring reinfection. ,FMT restores gut microbial balance, alleviates colonic and placental inflammation, and improves fetal growth. The specific correlations we identified (e.g., Ligilactobacillus with IPA/taurine, Desulfovibrio with arginine/IPA) provide mechanistic hypotheses for how specific microbes mediate these effects. In summary, these findings provide novel mechanistic insights into the gut-placenta axis in malaria, involving specific alterations in gut microbes and metabolites and highlight the potential of FMT as an experimental strategy for managing malaria in pregnancy.

Reviewer #2: This manuscript explores the interactions between gut health and malaria infection in a pregnant mouse model. The experimental work is extensive and comprehensive, and shows clearly that infection impacts gut health and the microbiota, that removal of the gut microbiota with antibiotic treatment impacts gut integrity (without impacting fetal outcomes) and transfaunation of gut microbiota improves gut health, reduces IFN-gamma production, and improves postnatal growth of pups. More methodological detail, reassessment of statistical analyses and a broader consideration of the results will improve the manuscript.

Response: Thank you for your positive assessment of our work and constructive suggestions. We have added more comprehensive methodological details in the materials and methods section; carefully reassessed all statistical analyses and results.. We believe these revisions have significantly improved the manuscript. All changes have been highlighted in the revised manuscript.

Reviewer #3: Strengths

The study employs a comprehensive approach, combining gut microbiota analysis, metabolomics, and fecal microbiota transplantation experiments to elucidate the role of gut microbiota in malaria-infected pregnant mice.

The use of multiple experimental groups and the inclusion of both early and late stages of infection help to provide a more complete picture of the impact of PbA infection on the gut-placenta axis.

Weaknesses

Clinical Relevance: While the study provides valuable insights into the role of gut microbiota in malaria-infected pregnant mice, the relevance of these findings to human malaria infection remains uncertain. The authors should acknowledge this limitation and suggest future studies to validate the findings in human populations.

Mechanistic Insights: The study demonstrates that FMT can alleviate placental and colonic inflammation and improve fetal weight, but the underlying mechanisms by which the gut microbiota influences these outcomes are not fully elucidated. Further exploration of the specific pathways and molecules involved would strengthen the study.

Experimental Design: The study does not include a control group of non-pregnant malaria-infected mice, which would have helped to distinguish the effects of pregnancy on the gut microbiota and placental pathology in the context of malaria infection.

Sample Size and Statistical Power: The sample size appears to be relatively small, which may limit the statistical power of the study and the generalizability of the findings. The authors should provide a justification for the chosen sample size and consider whether a larger sample size would be necessary for future studies.

Data Analysis: The analysis of the gut microbiota composition and metabolomics data is comprehensive, but the integration of these datasets could be improved. A more detailed analysis of the relationships between specific gut microbiota changes and metabolite alterations would provide a clearer understanding of the mechanisms involved.

Response：Thank you for your positive assessment and suggestion. We have revised the manuscript accordingly.

Clinical Relevance: we have acknowledged this limitation in the discussion and proposed specific directions for future clinical validation studies as follow: "while this study provides valuable insights into the role of gut microbiota in malaria-infected pregnant mice, the relevance of these findings to human malaria infection remains uncertain. Furthermore, the highly specific experimental conditions (e.g., standardized housing and infection protocols) do not fully represent the complex environmental and immunological challenges (such as malnutrition or co-infections) in endemic human populations." line 599-605

Mechanistic Insights: This study employed integrated multi-omics analysis to investigate the potential mechanisms. Our integrated analysis reveals that malaria-induced dysbiosis drives pathology through the gut-placenta axis:

1.Barrier Disruption: malaria-induced dysbiosis disrupts intestinal barrier function and intestinal barrier integrity (evidenced by reduced claudin-1/occludin) and increases pro-inflammatory cytokines (IFN-γ/TNF-α).

2.Metabolic Reprogramming: Serum metabolomics revealed significantly reduced levels of nutritional substrates such as amino acids and fatty acids. The dysbiosis triggers a pro-inflammatory metabolic shift, characterized by the depletion of key protective metabolites, including indole-3-propionic acid (IPA) and taurine, which are crucial for maintaining mucosal integrity and reducing inflammation.

3.Placental Stress:The mRNA expression of placental nutrient transporters—specifically the amino acid transporter Slc7a5, glucose transporter Slc2a1, and fatty acid transporter Fabp3—was upregulated. Concurrently, serum nutrient levels were reduced, suggesting a state of nutrient stress which may contribute to fetal growth restriction. These observations suggest that under conditions of insufficient maternal nutrient supply, the placenta may upregulate transporter expression to sustain nutrient acquisition.

Critically, microbiota transplantation (FMT) restores gut microbial balance, alleviates colonic and placental inflammation, and improves fetal growth. The specific correlations we identified (e.g., Ligilactobacillus with IPA/taurine, Desulfovibrio with arginine/IPA) provide mechanistic hypotheses for how specific microbes mediate these effects. In summary, these findings provide novel mechanistic insights into the gut-placenta axis in malaria, involving specific alterations in gut microbes and metabolites and highlight the potential of FMT as an experimental strategy for managing malaria in pregnancy.

Experimental Design: in order to distinguish the effects of pregnancy on the gut microbiota and placental pathology in the context of malaria infection, we had added a control group of non-pregnant malaria-infected mice. And then we performed 16S rRNA sequencing at 3 days (non-pregnant-PbD3) and 5 days (non-pregnant-PbD5) post-infection, respectively. Compared to the pregnant infected group (PbD3 and PbD5), there was no significant difference in the relative abundance of the phylum and genus levels, respectively (Supplementary Figure S3). Given that the placenta is a pregnancy-specific organ, assessment of placental pathology was appropriately confined to the pregnant groups, with uninfected pregnant mice serving as the relevant controls. This key finding indicates that pregnancy status itself did not markedly alter the core microbial shifts induced by PbA infection in our model, thereby strengthening the focus of our subsequent mechanistic investigations on the pregnant host.

Sample Size and Statistical Power: We had added this limitation in the discussion. “ First, the experimental group sizes were determined based on our preliminary studies and the ethical standards for pregnant animal experimentation to minimize animal suffering. However, the relatively small sample size in experimental groups may limit the statistical power and the generalizability of the findings, requiring larger cohorts for future studies.”

Data Analysis: We have enhanced the integration of microbiota and metabolomics data through additional correlation analyses and network visualizations. We had added and highlighted in the results and discussion of revised manuscript. line 473-490, line 568-586

Part II – Major Issues: Key Experiments Required for Acceptance

Reviewer #1: There are several occasions in which the authors overstate their conclusions. These include but are not limited to the following:

1) Line 39-41 – Overstatement to suggest FMT holds promise as strategy for managing pregnancy malaria.

Response: Thank you for your careful review. We apologized for our mistakes and revised to “Fecal microbiota transplantation (FMT) restores gut microbial balance, alleviates colonic and placental inflammation, and improves fetal growth.”line 35-37

2) Line 415 – Data do not evaluate pregnancy outcomes

Response: Thank you for your careful review. We apologized for our mistakes and revised as:

“Although no significant differences in parasitemia or pregnancy outcomes (fetal weights) were observed between the IN and IN-AB groups (Figure 6A-6C), the IN-AB group exhibited lower serum levels of FITC-dextran (Figure 6D), significant downregulation of colonic tight junction proteins (Claudin-1 and Occludin; Figure 6E), and elevated IFN-γ and TNF-α mRNA levels (Figure 6F).” line 499-503

3) Line 439 – “notable colonic inflammation” is overstated as only IFN-g is different.

Response: We apologized for our mistakes and revised to “Furthermore, FMT reduced the mRNA expression leve of proinflammatory cytokine IFN-γin colonic tissue (Figure 7J).”

4) Line 47-48 – Recommend using updated numbers from the WHO.

Response: We revised to “It is estimated 263 million clinical cases and approximately 597,000 deaths in 2023.” line 70-71

5) Introduction – The authors have failed to provide an appropriate summary of the previous work by Ref 2 in regards to gut microbiota-pregnancy malaria outcomes in mice.

Response: Thank you for your careful review. We added a summary of studies on the role of mice gut microbiota in pregnancy-associated malaria outcomes.

“Gut microbiota dysregulation has been implicated in pregnancy complications and adverse fetal outcomes through the gut-placenta axis (15). Smith et al. (2) demonstrates that gut microbiota composition correlates with malaria susceptibility in pregnancy and identified specific microbial taxa associated with infection outcomes. which remains primarily focused on establishing these correlations between microbial abundance and parasitemia. However, the gut microbiota influences fetal development, intestinal integrity, and host metabolism via the gut-placenta axis remain incompletely elucidated.” line 101-108

6) Figure 1, 6, and 7 – the authors should demonstrate that the changes in tight junction proteins results in decreased intestinal barrier integrity. One approach would be to gavage mice with FITC-dextran and assess FITC-dextral levels in the circulations.

Response: We performed FITC-dextran gavage assays to assess intestinal barrier integrity. The results are incorporated into Figures 2, 6, and 7.

Material method:

"Intestinal permeability was evaluated using fluorescein isothiocyanate (FITC)-dextran (24). Mice underwent fasting for 12 h and were administered FITC-dextran (50 mg/kg) via oral gavage. Blood was collected from the tail vein and centrifuged (12,000g, 5 min, 4℃). The serum FITC-dextran concentration was measured. A standard curve was generated using diluted FITC-dextran in nontreated serum diluted with PBS.”line 222-227

Results: "Notably, compared to PbD0 controls, the PbD3 and PbD5 groups showed significantly higher serum levels of FITC-dextran, which indicating loss of barrier integrity(Figure 2D)." line 403-405

Compared to the IN group, the IN-FMT and IN-AB groups showed significantly lower serum levels of FITC-dextran, respectively. (Figure 6D and Figure 7D).”

7) Figure 6.

o Details of antibiotic treatment days are not clear nor when the mice were harvested for subsequent analysis.

o There is no evidence that antibiotic treatment altered gut bacteria abundance or composition.

o Not clear how the antibiotic treatment in which most gut bacteria are eliminated is informative towards P. berghei ANKA-induced disruptions in gut bacteria on pregnancy outcomes.

Response: Thank you for your careful review. We apologized for the incomplete experimental information.

To determine the consequences of antibiotic treatment on host immune responses, intestinal barrier function and fetal health, we depleted gut microbiota using antibiotic treatment in PbA-infected pregnant mice.

“For depletion of the microbiota, mice were treated with a broad spectrum antibiotic mixture (ABX) consisting 100 mg/kg vancomycin, 200 mg/kg neomycin sulfate, 200 mg/kg metronidazole and 200 mg/kg ampicillin by oral gavage for 3 days from G8 to G11 in the IN-AB group (20). A fresh antibiotic solution was prepared daily to ensure activity. Control animals were not antibiotic treated. ”line 172-176

Secondly, we added Absolute Bacterial Quantification

“As previously described (25), each mouse was placed in a sterilized cage on the morning. Fresh fecal pellets were promptly collected and transferred to a pre-sterilized Eppendorf tube to prevent urine contamination. The fecal samples were immediately frozen in liquid nitrogen and stored at −80℃ until use.

To quantify the bacterial load in fecal matter, samples were aseptically collected from mice post-antibiotic treatment. Microbial DNA extraction was conducted utilizing the QuantiFast SYBR Green PCR Kit (Biorad) in conjunction with universal 16S rRNA primers (5′-ACTCCTACGGGAGGCAGCAG-3′ and 5′-ATTACCGCGGCTGCTGG-3′). The total bacterial count was subsequently calculated and expressed as the number of bacteria per milligram of fecal sample (26). Plot a standard curve with the positive control's log concentration on the x-axis and Ct value on the y-axis. Use the test sample's Ct value on this curve to find its log DNA concentration and calculate the actual concentration.”line 250-262

The results showed that antibiotic treatment reduced the bacterial load in infected animals by approximately 30-fold, indicating that antibiotic administration effectively depletes the gut microbiota. Although no significant differences in parasitemia or pregnancy outcomes (fetal weights) were observed between the IN and IN-AB groups (Figure 6A-6C), the IN-AB group exhibited lower serum levels of FITC-dextran (Figure 6D), significant downregulation of colonic tight junction proteins (Claudin-1 and Occludin; Figure 6E), and elevated IFN-γ and TNF-α mRNA levels (Figure 6F). Collectively, these results indicate that antibiotic treatment has no effect on parasitic infections or pregnancy outcomes (fetal weight) during PbA infection. While colonic damage and inflammation caused by PbA infection are dependent on the gut microbiota. Line 496-506

8) Figure 7 – the experiment would benefit from a vehicle control and gut microbiota analysis to demonstrate that the FMT changed the gut microbiota composition of treated animals.

Response: A PBS-gavaged control group with infection was indeed included in the experimental design as a vehicle control to account for potential effects of the gavage procedure itself. In Figure 7, we specifically chose to present a direct comparison between the IN and IN-FMT groups to clearly highlight the therapeutic effect of FMT. Consequently, the IN-PBS control omitted "PBS " in the particular figure panel to maintain clarity. We had added “As a vehicle control, mice in the infection (IN) group were gavaged with PBS. The experimental timeline is summarized in Supplementary Figure S1. ” in the method.

9) Figure 8

o Line 459-460 – were pups infected with P. berghei? The experimental plan of this data set is not clear.

Response: Thank you for your careful review. We apologized for the incomplete information. Indeed, some pups were infected with P. berghei. We supplemented it in materials and methods.

“On gestational day 18 (G18), six pregnant females were anesthetized using 2.5% Tribromoethanol, sacrificed by cervical dislocation and recorded fetal number and weight. In order to evaluate the offspring growing, the remaining pregnant mice (four per group in the UN, IN, and IN-FMT groups) were allowed to deliver naturally. Because infected dams exhibit compromised health, impaired lactation, disrupted maternal behavior, or even premature death postpartum-factors that can directly compromise offspring care and survival (27) (28). All newborns from deliver naturally were transferred to healthy surrogate mothers. Newborns were monitored and recorded body weights daily until weaning at postnatal day 20 (P20). And then, five offspring from each group (the UN, IN, and IN-FMT groups) were randomly selected, euthanized and measured colon lengths. While another five offspring from each group were re-infection with PbA to observe offspring survival rates.” line 317-328

o No statistics are provided for panel C or panel D.

Response: We added the requested statistical analysis to Panel C. Panel D presents survival data, which evaluates the entire survival distribution between groups over time. It is not customary to provide separate statistical annotations for individual time points. Our results showed that offspring from healthy dams (UN group) all dead at 11 post-reinfection days, whereas offspring from malaria-infected dams (IN group) showed a tendency toward prolonged survival after reinfection. However, the sample size in offspring reinfection experiment is limited. Therefore, this finding should be interpreted as a preliminary observation, and future studies with larger cohort sizes are warranted to substantiate this trend.

o Experimental set up is not clear and data do not make sense. It seems shocking that no pups survive beyond day 11 in the untreated group. Additionally, if no pups survive beyond day 11 in the untreated group, how is there weight measurement for this group through day 20?

Response: Thank you for your careful review. We apologized for the lack of clarity in our initial description. “On gestational day 18 (G18), six pregnant females were anesthetized using 2.5% Tribromoethanol, sacrificed by cervical dislocation and recorded fetal number and weight. In order to evaluate the offspring growing, the remaining pregnant mice (four per group in the UN, IN, and IN-FMT groups) were allowed to deliver naturally. Because infected dams exhibit compromised health, impaired lactation, disrupted maternal behavior, or even premature death postpartum-factors that can directly compromise offspring care and survival (27) (28). All newborns from deliver naturally were transferred to healthy surrogate mothers. Newborns were monitored and recorded body weights daily until weaning at postnatal day 20 (P20). And then, five offspring from each group (the UN, IN, and IN-FMT groups) were randomly selected, euthanized and measured colon lengths. While another five offspring from each group were re-infection with PbA to observe offspring survival rates.” line 317-328

Therefore, the body weight data were collected from naturally delivered offspring monitored from birth until weaning at 20 days. Whereas, the survival data were obtained from offspring with re-infection after weaned 20 days in the UN, IN, and IN-FMT groups.

Reviewer #2: The statistical analysis subsection of the methods section mentions statistical comparisons being done with students t test but this is not appropriate to analyses with three groups. Figure legends should specify statistical tests applied.

Response: Thank you for this critical observation regarding the statistical analysis. We apologized for the oversight in the Methods section. “For all comparisons across the three experimental groups , the data were analyzed using the non-parametric Kruskal-Wallis test.The threshold for statistical significance was established at P < 0.05.” line 361-363

Does Fig 5 correct for false discovery rate/multiple hypothesis testing?

Response: Thank you for your careful review. We did not perform false discovery rate (FDR) or multiple hypothesis testing correction for the correlation analysis in Fig 5, as the analysis involves a limited number of variables and serves as an exploratory analysis to identify potential associations. It is not a confirmatory test requiring strict control of multiple testing errors. Additiotonally, the sample size in this study is relatively small, and over-correction may increase the risk of false negatives in capturing true biological correlations.

Statements at lines 409-416 are not fully supported by the data shown in Fig 6.

Response: Thank you for your careful review. We revised the results description of Fig 6 as:

“To assess the impact of gut microbiota depletion on host immunity, intestinal barrier function, and fetal health, we treated PbA-infected pregnant mice with broad-spectrum antibiotics. The results showed that antibiotic treatment reduced the bacterial load in infected animals by approximately 30-fold, indicating that antibiotic administration effectively depletes the gut microbiota (Supplementary Figure S2). Although no significant differences in parasitemia or pregnancy outcomes (fetal weights) were observed between the IN and IN-AB groups (Figure 6A-6C), the IN-AB group exhibited lower serum levels of FITC-dextran (Figure 6D), significant downregulation of colonic tight junction proteins (Claudin-1 and Occludin; Figure 6E), and elevated IFN-γ and TNF-α mRNA levels (Figure 6F). Collectively, these results indicate that antibiotic treatment has no effect on parasitic infections or pregnancy outcomes (fetal weight) during infection. While colonic damage and inflammation caused by PbA infection are dependent on the gut microbiota.” line 494-506

Without more details regarding the timing of ABX treatment/transfaunation/FMT, it is difficult for the reader to interpret Figure 7

Response: Thank you for your careful review. We apologized for the incomplete experimental information and revised:.

“Antibiotic treatment and Fecal microbiota transplantation

For depletion of the microbiota, mice were treated with a broad spectrum antibiotic mixture (ABX) consisting 100 mg/kg vancomycin, 200 mg/kg neomycin sulfate, 200 mg/kg metronidazole and 200 mg/kg ampicillin by oral gavage for 3 days from G8 to G11 in the IN-AB group (20). A fresh antibiotic solution was prepared daily to ensure activity. Control animals were not antibiotic treated.” line171-176

“FMT was performed daily starting from G13 to G18 in the IN-FMT group. To mitigate the potential risk of pathogen transmission during FMT and ensure experimental reliability, the following quality control measures were implemented in this study: All donor mice were sourced from a Specific Pathogen Free - grade facility and were pre-screened for common murine pathogens, and only healthy individuals without signs of gastrointestinal infection were selected; the prepared bacterial suspensions were tested via both aerobic and anaerobic culture prior to transplantation to confirm the absence of exogenous pathogenic contamination; all procedures were performed within a biosafety cabinet using sterile instruments to avoid cross-contamination (22, 23). All mice were maintained under identical feeding and environmental conditions to ensure consistency. Their body weights were monitored weekly throughout the treatment period. As a vehicle control, mice in the infection (IN) group were gavaged with PBS.”line 185-197

Individual fetus weights are not independent, as they are influenced by the intrauterine environment, including number of total fetuses present (per dam). Statistical analysis (Fig 8B) must take into account this lack of independence.

Response: Thank you for your careful review. We acknowledged that fetal weights within the same litter are not statistically independent. While our analysis confirmed no significant difference in litter sizes across experimental groups (approximately 6 pups per litter). Thus, this factor minimizes potential confounding effects from variable litter sizes on fetal weight comparisons. We had supplemented the data on the number of fetal mice in supplementary figure S4.

The manuscript provides no methodological details of the data shown in Figure 8D-F.

Response: Thank you for your careful review. We apologized for the incomplete experimental information. We had revised the method details of the data shown in Figure 8D-F about FMT experiment.

“Antibiotic treatment and Fecal microbiota transplantation

For depletion of the microbiota, mice were treated with a broad spectrum antibiotic mixture (ABX) consisting 100 mg/kg vancomycin, 200 mg/kg neomycin sulfate, 200 mg/kg metronidazole and 200 mg/kg ampicillin by oral gavage for 3 days from G8 to G11 in the IN-AB group (20). A fresh antibiotic solution was prepared daily to ensure activity. Control animals were not antibiotic treated.

To prepare FMT material, fresh fecal samples from healthy gestation-phase BALB/c mice were collected daily into sterile cryovials. Aliquots (100 mg, 5-6 pellets) were immediately placed in ice-cooled containers. Processing involved homogenization with chilled PBS (1:10 w/v), 15-min 4℃ incubation, and 70 μm membrane filtration. Centrifugation (4℃, 5 min) yielded bacterial pellets resuspended in PBS to 1×1011CFU/L for transplantation. Prepared inocula were delivered to murine recipients through standardized oral gavage procedures at a dosage of 10 milliliters per kilogram body weight utilizing precision feeding apparatus (21).

FMT was performed daily starting from G13 to G18 in the IN-FMT group. To mitigate the potential risk of pathogen transmission during FMT and ensure experimental reliability, the following quality control measures were implemented in this study: All donor mice were sourced from a Specific Pathogen Free - grade facility and were pre-screened for common murine pathogens, and only healthy individuals without signs of gastrointestinal infection were selected; the prepared bacterial suspensions were tested via both aerobic and anaerobic culture prior to transplantation to confirm the absence of exogenous pathogenic contamination; all procedures were performed within a biosafety cabinet using sterile instruments to avoid cross-contamination (22, 23). All mice were maintained under identical feeding and environmental conditions to ensure consistency. Their body weights were monitored weekly throughout the treatment period. As a vehicle control, mice in the infection (IN) group were gavaged with PBS.” line 171-197

Reviewer #3: What metabolites have undergone significant changes and what roles do they play?

Response: Thank you for this question. Our integrated multi-omics analysis revealed significant alterations in metabolites and metabolic pathways, which are closely linked to the observed gut microbiota dysbiosis and might play a role in the disease pathogenesis. As detailed in the Results section, pathway enrichment analysis highlighted widespread dysregulation in: Taurine and hypotaurine metabolism.Valine, leucine, and isoleucine biosynthesis, Pyrimidine metabolism (essential for nucleic acid synthesis and cell proliferation), Pantothenate and CoA biosynthesis. Furthermore, our untargeted metabolomics identified several key metabolites from the prominently enriched amino acid metabolism pathways. Correlation Analysis between bacterial genera and differential metabolites were added. The functional implications of these metabolic changes are elaborated in the Discussion section. Line 473-490

Our integrated analysis revealed that PbA infection induces significant alterations in key serum metabolites, most notably the reduction of taurine, indole-3-propionic acid (IPA), and arginine, alongside perturbations in alanine and beta-alanine. These changes are critically important as they are closely correlated with specific microbial shifts (e.g., depletion of Ligilactobacillus and expansion of Desulfovibrio) and are mechanistically linked to disease pathogenesis. Specifically, the decrease in taurine and IPA, both crucial for maintaining mucosal integrity and exerting anti-inflammatory effects, likely contributes to the breakdown of the gut barrier and exacerbation of inflammation. Concurrently, the reduction in arginine, an amino acid essential for cellular repair and anti-inflammatory responses, further compromises the host's ability to mitigate tissue damage. The metabolic dysregulation across these pathways might participate in impaired barrier function, reduced capacity for tissue repair, and heightened inflammatory responses. Line 473-490

Part III – Minor Issues: Editorial and Data Presentation Modifications

Please use this section for editorial suggestions as well as relatively minor modifications of existing data that would enhance clarity.

Reviewer #1: Grammer and sentence structure should be carefully reviewed throughout the text.

Response: Thank you for your careful review. We had carefully reviewed grammar and sentence structure.

Reviewer #2: Methods

Provide more details regarding how fostering of newborn pups to surrogate dams was performed.

Response: Thank you for your careful review. We had added the details regarding how fostering of newborn pups to surrogate dam:

“On gestational day 18 (G18), six pregnant females were anesthetized using 2.5% Tribromoethanol, sacrificed by cervical dislocation and recorded fetal number and weight. In order to evaluate the offspring growing, the remaining pregnant mice (four per group in the UN, IN, and IN-FMT groups) were allowed to deliver naturally. Because infected dams exhibit compromised health, impaired lactation, disrupted maternal behavior, or even premature death postpartum-factors that can directly compromise offspring care and survival (27) (28). All newborns from deliver naturally were transferred to healthy surrogate mothers. Newborns were monitored and recorded body weights daily until weaning at postnatal day 20 (P20). And then, five offspring from each group (the UN, IN, and IN-FMT groups) were randomly selected, euthanized and measured colon lengths. While another five offspring from each group were re-infection with PbA to observe offspring survival rates. ”Line 317-328

How was gut microbial depletion confirmed? What was the timing used for ABX treatment and transfaunation of gut microbiota in relation to initiation of pregnancy and PbA infection? Were FMT mice ABX treated first or just received an FMT? Was the FMT material from pregnant mice?

Response: Thank you for your careful review. We apologize for the incomplete experimental information. We had revised and added the experimental timeline is summarized in Supplementary Figure S1.

For depletion of the microbiota, mice were treated with a mixture of ABX (100 mg/kg vancomycin, 200 mg/kg neomycin sulfate, 200 mg/kg metronidazole and 200 mg/kg ampicillin) by oral gavage for 3 days from G8 to G11 [1]. A fresh antibiotic solution was prepared daily to ensure activity. Control animals were not antibiotic treated. Line 172-176

In addition, we had added Absolute Bacterial Quantification

To quantify the bacterial load in fecal matter, samples were aseptically collected from mice post-antibiotic treatment. Microbial DNA extraction was conducted utilizing the QuantiFast SYBR Green PCR Kit (Biorad) in conjunction with universal 16S rRNA primers (5′-ACTCCTACGGGAGGCAGCAG-3′ and 5′-ATTACCGCGGCTGCTGG-3′). The total bacterial count was subsequently calculated and expressed as the number of bacteria per milligram of fecal sample[1]. Plot a standard curve with the positive control's log concentration on the x-axis and Ct value on the y-axis. Use the test sample's Ct value on this curve to find its log DNA concentration and calculate the actual concentration. Line 254-262

The results showed that antibiotic treatment reduced the bacterial load in infected animals by approximately 30-fold, indicating that antibiotic administration effectively depletes the gut microbiota.

FMT mice just received an FMT treatment with out ABX treatment. To prepare FMT material, fresh fecal samples from healthy gestation-phase BALB/c mice were collected daily into sterile cryovials. Aliquots (100 mg, 5-6 pellets) were immediately placed in ice-cooled containers. Processing involved homogenization with chilled PBS (1:10 w/v), 15-min 4℃ incubation, and 70 μm membrane filtration. Centrifugation (4℃, 5 min) yielded bacterial pellets resuspended in PBS to 1×1011CFU/L for transplantation. Prepared inocula were delivered to murine recipients through standardized oral gavage procedures at a dosage of 10 milliliters per kilogram body weight utilizing precision feeding apparatus.

FMT was performed daily starting from G13 to G18 in the IN-FMT group. To mitigate the potential risk of pathogen transmission during FMT and ensure experimental reliability, the following quality control measures were implemented in this study: All donor mice were sourced from a Specific Pathogen Free - grade facility and were pre-screened for common murine pathogens, and only healthy individuals without signs of gastrointestinal infection were selected; the prepared bacterial suspensions were tested via both aerobic and anaerobic culture prior to transplantation to confirm the absence of exogenous pathogenic contamination; all procedures were performed within a biosafety cabinet using sterile instruments to avoid cross-contamination (22, 23). All mice were maintained under identical feeding and environmental conditions to ensure consistency. Their body weights were monitored weekly throughout the treatment period. As a vehicle control, mice in the infection (IN) group were gavaged with PBS. The experimental timeline is summarized in Supplementary Figure S1. Line 177-198

[1]	Chen X, Wu R, Li L, Zeng Y, Chen J, Wei M, et al. Pregnancy-induced changes to the gut microbiota drive macrophage pyroptosis and exacerbate septic inflammation. Immunity. 2023;56(2):336-52.e9.

Results

Figure resolution is low throughout, making it difficult to evaluate.

Response: Thank you for your careful review. We had uploaded each picture separately.

Line 303: provide reference(s)

Response: we had provided reference.

Aramys S Reis,Renato Barboza,Oscar Murillo. Inflammasome activation and IL-1 signaling during placental malaria induce poor pregnancy outcomes. Sci Adv,2020 Mar 4;6(10):eaax6346. doi: 10.1126/sciadv.aax6346. eCollection 2020 Mar.

However, we had deleted this sentence based on another review. Therefore, we had deleted this reference.

Samples sizes included in each figure/panel should be provided in the legend unless it is obvious (ie Fig 3 scatter plots or individual microbiota compositions).

Response: We added the sample size in the figure legend.

Check typo at lines 346-7, re Fig 3E

Response: We revised“Fig 3D and 3E”

Lines 348-350: why are only two of the highlighted genera shown in Figure 3F?

Response: Thank you for your careful review. In Figure 3F, we specifically highlighted Ligilactobacillus and Desulfovibrio because they exhibited the most statistically and biologically significant changes among beneficial and harmful bacteria, respectively. By focusing on these two representative genera—which displayed the greatest magnitude of change—we aimed to concisely illustrate the microbiota shift’s key trends. Ligilactobacillus and Desulfovibrio were selected as key genera based on their representative functions in the gut microbiota and their established links to host health. As a beneficial bacterium, an increase in the abundance of Ligilactobacillus typically indicates a favorable metabolic and immune status, as it strengthens the intestinal barrier and suppresses inflammation through the production of short-chain fatty acids. In contrast, Desulfovibrio, a sulfate-reducing bacterium often regarded as a harmful taxon, can lead to elevated hydrogen sulfide levels when its abundance rises. This metabolite is directly toxic to intestinal epithelial cells, compromising the mucosal barrier and promoting systemic inflammatory responses—a pathophysiology highly relevant to disease states such as malaria infection.

Section starting at line 351: Not sure it is appropriate to refer to differentially abundant genera as “biomarkers”

Response: We revised to “Differentially abundant microbial genera identified by LEfSe analysis”

What are the y axis units for Fig 4G?

Response: Thank you for your careful review. A comprehensive screening of metabolites was performed using untargeted metabolomics. Untargeted metabolomics, with its unbiased and global detection profile, is a key discovery tool for novel metabolites and biological mechanisms, with a workflow focused on inter-group differences that naturally yields results as relative abundances [1,2]. The Y-axis indicates the relative levels of metabolites across groups measured by untargeted metabolomics (Fig 4G).

We have added it in the figure legend “(G) The Y-axis represents the relative content of the corresponding metabolites between each group..”

[1]Chen L, Lu W, Wang L, et al. Metabolite discovery through global annotation of untargeted metabolomics data. Nat Methods. 2021;18(11):1377-1385. doi:10.1038/s41592-021-01303-3

[2]Schrimpe-Rutledge AC, Codreanu SG, Sherrod SD, McLean JA. Untargeted Metabolomics Strategies-Challenges and Emerging Directions. J Am Soc Mass Spectrom. 2016;27(12):1897-1905. doi:10.1007/s13361-016-1469-y

Statement at lines 248-430 is not supported by the data show in Fig 7.

Response: Thank you for your careful review. We had revised the description about the Fig 7 as:

“To investigate whether restoring microbial homeostasis could mitigate infection-induced pathology, FMT from healthy donors markedly ameliorated PbA-induced colonic pathology in pregnant mice. The IN-FMT group showed decreased in parasitemia and significantly incresed vascular spaces (Figure 7A and 7B). There was no significant differences in the length of the colon between two groups (Figure 7C). However, compared to he IN group, the IN-FMT group exhibited significant structural recovery of the colonic mucosa, including orderly and denser arrangement of intestinal villi, regular morphology of intestinal crypts, along with a reduction in inflammatory cell infiltration and increased crypt depth (Figure 7E, 7F). Compared to IN group, the IN-FMT group showed significantly lower serum levels of FITC-dextran (Figure 7D). FMT intervention attenuated the infection-induced impairment of intestinal tight junctions, as evidenced by the elevated expression of ZO-1 protein (Figure 7G and 7H) and upregulation of ZO-1, Claudin-1, and Occludin mRNA levels (Figure 7I). Furthermore, FMT also reduced the mRNA expression leve of proinflammatory cytokine IFN-γin colonic tissue (Figure 7J).” line510-524

Figure 7D requires more annotation to support the statement made at lines 431-433.

Response: we had added more annotation of Figure 7D as: Compared to he IN group, the IN-FMT group exhibited significant structural recovery of the colonic mucosa, including orderly and denser arrangement of intestinal villi, regular morphology of intestinal crypts, along with a reduction in inflammatory cell infiltration and increased crypt depth (the new Figure 7E and 7F) . line 515-518

The data shown in Fig S1 are important and their incorporation into the body of the manuscript is recommended.

Response: Thank you for your careful review. We had incorporation into the body of the manuscript as Fig 9.

Discussion

The results with ABX and FMT were mixed (less inflammation but reduced crypt length with FMT; with ABX alone, reduced tight junction protein expression but increased inflammation). More discussion is required to interpret these results

Response：Thank you for the insightful comments. We apologized that we had mistake the labels for the IN group and the IN-FMT group during typesetting. We have corrected the figure. The results showed that “ However, compared to he IN group, the IN-FMT group exhibited significant structural recovery of the colonic mucosa, including orderly and denser arrangement of intestinal villi, regular morphology of intestinal crypts, along with a reduction in inflammatory cell infiltration and increased crypt depth (Figure 7E, 7F).” line 515-518

Line 509: what is meant by “prolonged” PbA infection?

Response: Thank you for your careful review. The term "prolonged" was used to generally describe the progression of infection from the earlier (PbD3) to the later (PbD5) time point. To improve accuracy, we have removed "prolonged" from the manuscript.

Reviewer #3:

Title and Abstract:

The title is appropriate and concise, accurately reflecting the core content of the study. However, the abstract should better highlight the innovative aspects of the gut microbiome research in malaria-infected pregnant mice and its clinical implications. For example, the specific changes in gut microbiota and their direct impact on pregnancy outcomes could be emphasized.

Response: Thank you very much for your valuable comments. We have revised the abstract section “Malaria in pregnancy remains a major global health concern, contributing significantly to maternal and fetal morbidity and mortality. While gut microbiota dysregulation has been implicated in pregnancy complications and malaria pathogenesis, its functional role and the underlying mechanisms within the gut-placenta axis during placental malaria are poorly understood. In pregnant mice, Plasmodium berghei ANKA infection disrupts the gut-placenta axis, leading to intestinal inflammation, placental injury and reduced fetal weight. Microbiome analysis revealed gut dysbiosis characterized by reduced Ligilactobacillus and increased Desulfovibrio. The metabolomic profiling identified disruption of amino acid and fatty acid metabolism, including indole-3-propionic acid and taurine. These microbial and metabolic alterations may contribute to impaired intestinal barrier integrity and dysregulated inflammatory responses. Critically, fecal microbiota transplantation (FMT) restores gut microbial balance, alleviates colonic and placental inflammation, and improves fetal growth. These findings provide novel mechanistic insights into the gut-placenta axis in malaria and highlight the potential of FMT as a experimental strategy for managing malaria in pregnancy. Future studies should validate these findings in clinical settings and explore alternative microbiota-targeted interventions.” line 24-41

Introduction:

The background introduction is comprehensive, but it could more clearly establish the connection between gut microbiota and malaria infection in pregnant mice. The current explanation remain unclear. For instance, when discussing the impact of malaria on pregnant women, it could specifically mention how these effects are linked to gut microbiota alterations. For Malaria Data, please update to 2023.

The literature review could be more focused. While citing numerous studies on malaria and gut microbiota, it should better integrate them with the current research objectives. For example, after introducing previous findings on gut microbiota changes in malaria patients, it should explicitly state the gaps these studies leave and how this study addresses them.

Response: Thank you very much for your valuable comments. We have updated Malaria Data to 2023. Additionally, we had added the impact of malaria on pregnant women, as well as explicitly state the gaps these studies leave and how this study addresses them.

“The gut microbiota, comprising diverse microbial communities in the gastrointestinal tract, plays a critical role in maintaining intestinal barrier integrity and regulating immune responses (11, 12). Recent research has indicated that imbalances or dysregulation in gut microbiota composition are associated with exacerbation of inflammation and increased susceptibility to infections during malaria (13) (14). However, existing studies on the gut microbiota in malaria has primarily focused on non-pregnant models or clinical observations of microbial shifts. Consequently, the functional implications of gut microbiota homeostasis during Plasmodium infection in pregnant hosts remains poorly understood.

Gut microbiota dysregulation has been implicated in pregnancy complications and adverse fetal outcomes through the gut-placenta axis (15). Smith et al. (2) demonstrates that gut microbiota composition correlates with malaria susceptibility in pregnancy and identified specific microbial taxa associated with infection outcomes. which remains primarily focused on establishing these correlations between microbial abundance and parasitemia. However, the gut microbiota influences fetal development, intestinal integrity, and host metabolism via the gut-placenta axis remain incompletely elucidated.

In this study, pregnant mice were intravenously infected with Plasmodium berghei ANKA (PbA), resulting in placental damage, intestinal inflammation, adverse pregnancy outcomes and disruption of the gut-placenta axis. The composition of gut microbiota in fecal samples was assessed using the Illumina MiSeq platform, and serum metabolite levels were analyzed through LC-MS/MS-based metabolomics. The functional role of the gut microbiota was further investigated through fecal microbiota transplantation (FMT) experiments. Additionally, we evaluated the transgenerational impact of maternal infection by assessing the growth and reinfection susceptibility of the offspring. Collectively, our findings delineate how the gut microbiota influences pregnancy outcomes by modulating intestinal barrier integrity, host metabolism, and inflammatory responses, thereby providing deeper mechanistic insights into its role in gestational malaria pathogenesis.” line 92-120

Methods:

In the animal experiment section, the grouping and infection procedures are clearly described. However, the criteria for selecting the specific dose and timing of infection could be explained. For example, why was day 13 of gestation chosen for infection? What is the basis for using an intravenous inoculation of 1×106 PbA-infected erythrocytes? This information would help readers better understand the experimental design rationale.

For the FMT experiment, the preparation and transplantation process of fecal microbiota is detailed. Nevertheless, the potential risks and quality control measures of FMT, such as how to avoid the transmission of pathogens, could be mentioned to enhance the reliability of the experimental methods.

Response: Thank the reviewers for their insightful suggestions, which have significantly improved our manuscript. We have addressed all concerns as detailed below.

Point 1: we had provided the criteria for selecting the specific dose and timing of infection in the revised Methods section.

“Based on the preliminary studies demonstrating that earlier administration (G1-10) resulted in pregnancy termination prior to fetal viability, the timing of infection was scheduled for gestational day 13 (G13) (17). As to the infection doses, it was reported that lower inoculum doses (≤1×105) failed to establish effective infection models, whereas higher doses (≥1×107) induced acute lethal responses in hosts (18, 19). The dosage parameter (1×10^6^) ensures could sustain parasitemia and maintain the stability of the pregnancy model. ” line 150-156

Point 2: As to the potential risks associated with FMT, we supplemented the Methods section with detailed quality control measures:

“To mitigate the potential risk of pathogen transmission during FMT and ensure experimental reliability, the following quality control measures were implemented in this study: All donor mice were sourced from a Specific Pathogen Free - grade facility and were pre-screened for common murine pathogens, and only healthy individuals without signs of gastrointestinal infection were selected; the prepared bacterial suspensions were tested via both aerobic and anaerobic culture prior to transplantation to confirm the absence of exogenous pathogenic contamination; all procedures were performed within a biosafety cabinet using sterile instruments to avoid cross-contamination (22, 23). All mice were maintained under identical feeding and environmental conditions to ensure consistency. Their body weights were monitored weekly throughout the treatment period. As a vehicle control, mice in the infection (IN) group were gavaged with PBS.” line 185-197

Results:

The results are rich in data, but the presentation could be more organized. For example, the histopathological changes of the placenta and colon, alterations in gut microbiota composition, and variations in serum metabolites could be grouped and discussed in a logical sequence to make it easier for readers to follow.

Some graphical representations of the results could be improved. For instance, in Figures 1–8, the labels and legends could be made clearer and more specific. In Figure 1, the representative images of H&E-stained placental tissue sections could indicate the specific regions being observed and the differences between groups. In Figure 2, the histopathological changes of colonic samples could provide a more detailed description of the inflammatory cell infiltration.

The correlation analysis between gut microbiota and serum metabolites is a key part of the results. However, the interpretation of the correlation coefficients and p-values in the correlation analysis results could be more in-depth. For example, what biological significance do the positive and negative correlations between specific gut microbiota and metabolites imply?

Response: We sincerely thank the reviewer for these suggestions, which have helped us significantly improve the clarity and depth of our Results section. We have addressed each point as follows:

Reorganization of Results:

As suggested, we have reorganized the flow of the Results section to present the findings in a more logical sequence. It begins with the histopathological changes in the placenta and colon, then introduces the gut microbiota alterations, and finally integrates these with the serum metabolomics data, thereby building a coherent story that culminates in the correlation analysis.

Improvement of Graphical Representations

We had added the more clear labels and legends in Figures 1-8 and revised the Figure Legends. We have revised Figure 1 accordingly to provide clearer indications of the specific histological structures and differences. Specific annotations have been added to the representative H&E-stained placental tissue sections: an asterisk (*) now denotes the maternal blood sinus, and a triangle (▲) denotes the trophoblast cells. We plotted a quantitative statistical chart to show the differences. Furthermore, we had expanded the description of the histopathological changes in the Results section with the following statement:

“As shown in Figure 1C, with the progression of P. berghei ANKA infection, morphological alterations were observed in the trophoblast cells, accompanied by a narrowing of the maternal blood sinuses. These changes resulted in a reduction of the space available for maternal-fetal exchange.” line 374-377

For the histopathological changes in the colonic samples shown in Figure 2, we provide a more detailed description of the inflammatory cell infiltration.

“As shown in the Figure 2D, HE staining of the colon tissue revealed that the morphological structure was characterized by an intact colonic mucosa, neatly arranged intestinal villi, clear crypt outlines, and an absence of pathological alterations such as edema or ulceration in the PbD0 group. While, significant structural damage of colonic tissue in the infection groups were characterized by irregular crypt morphology, atrophy of villi and crypts, disordered arrangement of goblet cells, and accompanying inflammatory cell infiltration. Furthermore, significant differences in colonic crypt depth were detected between the PbD5 group and the other two groups (Figure 2F).” line 392-399

In-depth Interpretation of Correlation Analysis:
To provide a deeper insight into the gut microbiota-metabolite correlations, we have made enhancements across three parts of the manuscript:

Method: “To explore potential relationships between fecal bacterial genera and serum differential metabolites, a Spearman’s rank correlation analysis was performed and and visualized by heatmap in R software using the “heatmap” package. The spearman rank correlation coefficients were provided to quantify these relationships and associations.” line 354-358

Results: “Notably, the relative abundance of Lactobacillus reuteri was positive correlated with indole-3-propionic acid (IPA) (r=0.53, p=0.0263) and taurine (r=0.57, p=0.0154), suggesting its role in maintaining these beneficial metabolites-an effective antioxidant and immunomodulator. Conversely, the relative abundance of Desulfovibrio shows significant negative correlations with arginine (r=-0.57, p=0.0158) and IPA (r=-0.62, p=0.0069), implieing the proliferation of Desulfovibrio may deplete arginine (an important amino acid involved in cell repair) or suppress IPA production. Collectively, these findings demonstrate a close relationship between changes in the gut microbiota and metabolites, suggesting a potential mechanism underlying infection-related metabolic imbalance.” line 481-490

Discussion: “ As a key probiotic, Ligilactobacillus contributes to intestinal homeostasis by enhancing the barrier function and suppressing inflammation. Ligilactobacillus depletion likely exacerbates endotoxemia-induced inflammation by weakening mucosal defenses (31). The decline in Ligilactobacillus is correlated with reduced levels of indole-3-propionic acid (IPA) and taurine, both crucial for mucosal integrity and anti-inflammatory responses. The loss of Ligilactobacillus may disrupt microbial collaboration in the gut, thereby hindering the production of IPA by other bacteria, such as Clostridium (34, 35), while also impairing competitive taurine utilization and its conversion into health-promoting metabolites (36,37). Conversely, the expansion of Desulfovibrio was associated with decreased arginine and IPA, which were essential for cellular repair and anti-inflammatory responses. Moreover, Desulfovibrio-derived hydrogen sulfide can directly damage the intestinal epithelium and promote inflammation (38, 39). These findings support the association of Desulfovibrio with metabolic imbalances and a loss of gut ecosystem stability (40, 41). ” line 572-586

We believe these revisions have greatly enhanced the organization, clarity, and scientific rigor of our results.

Discussion:

The discussion effectively links the results to the research objectives and hypotheses. However, it could more critically evaluate the limitations of the study. For example, apart from the limitation of using an animal model, other potential limitations such as the relatively small sample size and the specificity of the experimental conditions could be discussed.

When discussing the clinical implications of the findings, it could more explicitly suggest directions for future research. For instance, it could propose specific plans for clinical trials of FMT in pregnant women with malaria, including trial design, participant selection, and outcome indicators.

Response: We expanded the Discussion section to provide a more critical evaluation of the study's limitations and to outline more explicit and actionable plans for future research, as detailed below.

“These findings provide mechanistic insight into the role of the gut-placenta axis in malaria during pregnancy and highlight the potential of FMT, yet several limitations should be considered. First, the experimental group sizes were determined based on our preliminary studies and the ethical standards for pregnant animal experimentation to minimize animal suffering. However, the relatively small sample size in experimental groups may limit the statistical power and the generalizability of the findings, requiring larger cohorts for future studies. Second, while this study provides valuable insights into the role of gut microbiota in malaria-infected pregnant mice, the relevance of these findings to human malaria infection remains uncertain. Furthermore, the highly specific experimental conditions (e.g., standardized housing and infection protocols) do not fully represent the complex environmental and immunological challenges (such as malnutrition or co-infections) in endemic human populations. These findings will require validation through future clinical studies in human populations.

To address these limitations and advance the clinical implications of these findings, future research should prioritize well-designed clinical trials. A randomized, double-blind, placebo-controlled design would be recommended to rigorously evaluate the efficacy and safety of FMT as an adjunct to standard antimalarial therapy. Specifically, the trial design also needs to address: (1) stringent participant selection criteria, (2) optimized treatment protocols, and (3) comprehensive outcome measures, including maternal anemia, fetal growth parameters, and longitudinal microbiome profiling. Furthermore, exploring alternative microbiome-modulating strategies—such as targeted probiotics, prebiotics, or selective antibiotics—may offer more scalable and practical therapeutic avenues.” line 593-616
