# Supplementary material for: Fecal microbiota transplantation alleviates adverse pregnancy outcomes and intestinal injury in experimental malaria
Source: PLoS Pathog. 2026 Aug 20;22(8):e1014459. doi: 10.1371/journal.ppat.1014459 (PMC13492792; doi:10.1371/journal.ppat.1014459)
Supplement: Supplementary file 2 [file ppat.1014459.s010.docx]

Dear Pro.

Thank you for giving us the opportunity to submit a revised version of our manuscript, “Gut Microbiota Modulation in Malaria-Infected Pregnant mice: Implications for Maternal and Neonatal Health” (PPATHOGENS-D-25-00948), for consideration for publication in PLOS Pathogens. We also wish to extend our sincere gratitude to the reviewers for their time and insightful comments, which have been invaluable in helping us strengthen our work.

We have now carefully addressed all the points raised. Below, you will find our point-by-point responses to the reviewers’ comments. All corresponding changes have been incorporated into the manuscript, and the revised text has been highlighted for easy reference.

Jiangnan University Medical Center, Department of Public Health and Preventive Medicine, Wuxi School of Medicine, Jiangnan University, Wuxi, China

E-mail: woerseng@126.com

Thank you very much for your attention and consideration.

Best regards.

Yours sincerely,

Cheng yang

**Response to Reviewers**

Reviewers' Comments:

Part I - Summary
Please use this section to discuss strengths/weaknesses of study, novelty/significance, general execution and scholarship.

Reviewer #1:Han S, et al, have provided an extensively revised manuscript that addresses many of my prior concerns. I find this to be substantially improved manuscript that will be an important contribution to the malaria-gut microbiome research community. I do have several remaining concerns.

Respond: We sincerely thank the reviewer for the positive assessment of our revised manuscript. We have carefully addressed the remaining comments in detail below.

Reviewer #2:This revised manuscript examines how malaria infection in a pregnant mouse model impacts the gut microbiota, intestinal integrity, metabolism and offspring outcomes. The revision presents an improved manuscript, but some additional revisions are required to meet journal standards.

Respond: We thank the reviewer for the thoughtful evaluation of our revised manuscript. We have addressed each of the remaining points in detail below.

Part II – Major Issues: Key Experiments Required for Acceptance

Please use this section to detail the key new experiments or modifications of existing experiments that should beabsolutelyrequired to validate study conclusions.
Generally, there should be no more than 3 such required experiments or major modifications for a ”Major Revision” recommendation. If more than 3 experiments are necessary to validate the study conclusions, then you are encouraged to recommend ”Reject”.

**Reviewer #1:**

1. Line 499-494 – The data suggest a more nuanced interpretation than “While colonic damage and inflammation caused by PbA infection are dependent on the gut microbiota.” Following antibiotic-induced reduction in gut microbiota, there is improved intestinal barrier integrity (reduction in serum FITC-dextran) while reduced expression of claudin-1 and occluding. These latter results suggest reduced intestinal barrier integrity, which is consistent with increased pro-inflammatory cytokines, IFN-g and TNF. Instead of a broad, non-specific conclusion the authors can provide more specific interpretations and conclusions from these results.

Response: We sincerely thank the reviewer for this comment. FITC-dextran is a widely used indicator of intestinal barrier permeability. Increased FITC-dextran levels in serum reflect enhanced intestinal permeability and impaired barrier integrity.

We carefully re-examined the original experimental records and raw data. We found that the standard curve used for FITC-dextran quantification in that experimental batch was non-optimal, which may have affected the accuracy of the serum FITC-dextran concentrations. We repeated the FITC-dextran permeability assay using a newly established cohort of mice. The newly established FITC-dextran standard curve has been provided in the below.

Accordingly, the relevant Results and Discussion sections have been revised:

Results:“the IN-AB group exhibited higher serum levels of FITC-dextran (Figure 6D)” (Lines 511).

“Collectively, these results indicate that depletion of the gut microbiota did not affect parasitemia or pregnancy outcomes, but was associated with aggravated intestinal barrier dysfunction and colonic inflammation during PbA infection”(Lines 513- 516).

Discussion:“Furthermore, depletion of the gut microbiota by broad-spectrum antibiotics exacerbated PbA infection-induced intestinal injury,” (Lines 574-575).

| Fluorescence Intensity | FITC-dextran concentration （ug/mL)） |
| --- | --- |
| 2620 | 220.812 |
| 1018 | 82.7011 |
| 405 | 30.9742 |
| 179 | 11.6008 |
| 106 | 4.34488 |
| 82 | 1.62729 |
| 69 | 1.62729 |
| 55 | 0.609474 |

Reviewer #2:No new experiments are requested. However, some data reanalysis should be considered.
Methods
1.Methods used for pathological and quantitative evaluation of placenta and intestinal tissue (data shown in Figure 1, 2, 7) need to be described. Were the samples evaluated in a blinded fashion? By how many independent evaluators?

Response: Thank you for this important comment. For histopathological and quantitative analysis, placental and colonic sections were assessed using a combination of morphometric measurements and semi-quantitative scoring. All samples were evaluated by three independent evaluators in a blinded manner. Furthermore, placental structure was quantified by calculating the area ratios of the labyrinth and junction zones using ImageJ software. Colonic injury was evaluated based on histological features, crypt depth was quantitatively measured using ImageJ..

We have revised the manuscript as:

“Histopathological evaluation was independently evaluated by three blinded investigators. Quantitative and semi-quantitative analyses were performed using a combination of morphometric measurements and histological scoring. Furthermore, placental structure was quantified by calculating the area ratios of the labyrinth and junction zones using ImageJ software. Colonic injury was assessed based on histological features, and crypt depth was measured quantitatively using ImageJ.”(Line 208-213)

2.Evaluation of fetal and postnatal outcomes: Is it correct that n=2 dams per group?

Response: We thank the reviewer for this important comment. We agree that the number of dams included in this study was limited (n = 2). Owing to the inherent complexity of the pregnant malaria model, including infection-associated maternal mortality and reduced reproductive success, only two of the five infected dams remained available for subsequent offspring-related analyses. Although multiple offspring were obtained from each dam and five were selected for analysis, the effective biological sample size was determined by the number of dams, representing a limitation of this study.

We have acknowledged this limitation in the Discussion section:“Firstly, owing to the complexity of the malaria-infected pregnancy model, including infection-associated maternal mortality and reduced reproductive success, the number of dams available for offspring-related analyses was limited. Future studies incorporating larger cohorts and gestational age-matched controls are required, further to distinguish the effects of infection from those of pregnancy.”(Lines 614-620).

3.Statistics: Correlation analysis (Fig 5) must either control for multiple hypothesis testing with posthoc adjustments, or the limitations of failing to account for Type I error must be acknowledged in the methods and results sections. For groupwise comparisons, Kruskal-Wallis test was used with which posthoc test for pairwise comparisons?

Response: Thank you for your valuable comment. To control for multiple hypothesis testing in the microbiota–metabolite correlation analysis, P values were adjusted using the Holm–Šídák method. Additionally, among three groups, the Kruskal–Wallis test was used to assess overall group differences. Pairwise comparisons between two groups were conducted separately using the Mann–Whitney U test. The revised text is as follows:

Method: “To explore potential associations between differential bacterial genera and differential metabolites, Spearman's rank correlation analysis was performed using R software. Correlation coefficients (r) were calculated to evaluate the strength and direction of the associations. For comparisons among three groups, the Kruskal-Wallis test was used to assess overall group differences. Pairwise comparisons between two groups were conducted separately using the Mann-Whitney U test. To control for multiple hypothesis testing, P values were adjusted using the Holm-Šídák method, and an adjusted P < 0.05 was considered statistically significant. Significant correlations were visualized using a heatmap.”(Lines362-365, 368-371).

Result: “To explore the associations between differential bacterial genera and metabolites, Spearman's correlation analysis was performed and visualized as a heatmap (Figure 5A). Notably, the relative abundance of Monoglobus was positively correlated with LPC(22:6) (r=0.89, adjusted P < 0.05), suggesting a potential role for this genus in maintaining lipid metabolic homeostasis during infection. In contrast, the potentially pathogenic genus Desulfovibrio exhibited a strong negative correlation with 2-(2-(19-Acetamido-16,18-dihydroxy-5,9-dimethyl-6-oxoicosan-7-yl) oxy-2-oxoethyl) butanedioic acid (r=-0.86, adjusted P < 0.05), implying that the expansion of Desulfovibrio may contribute to the depletion of this metabolite during disease progression. In addition, Alistipes and Odoribacter respectively exhibited significant positive and negative correlations with multiple metabolites involved in lipid and nucleoside metabolism (|r| = 0.84-0.87, adjusted P < 0.05). Collectively, these results revealed significant associations between differential gut bacterial genera and host metabolites following PbA infection, highlighting coordinated alterations in the gut microbiota and metabolome..”(Lines488-502).

Results
1.Figure 1A, B, C, D compare mice across different gestation days when placental morphology would be expected to change. PbD3 (G16) and PbD5 (G18) data should be compared against uninfected pregnant mice at the same gestational age. Lines 155-157 indicate such mice were generated; their data should be shown in Fig 1. Without these critical control data, the results are difficult to interpret as the effects of advancing pregnancy cannot be distinguished from the effects of advancing infection.

Response: Thank the reviewer for this important comment. We completely agree that distinguishing normal gestational progression from infection-induced effects requires same-age uninfected controls. Accordingly, we have compared uninfected and infected pregnant mice at the corresponding gestational ages (G16 and G18). However, due to the limited number of panels in the main figures, we have presented these control comparisons in Supplementary Figure 2 rather than in the main figure. The result showed that compared to uninfected pregnant mice at the same gestational age, infected mice respectively showed enlarged labyrinth zones, reduced junction zones, and narrowed vascular spaces at both G16 and G18, with more pronounced alterations at G18.

We have revised the result as: “To distinguish the effects of PbA infection from normal gestational progression, we compared placental histopathology between infected and uninfected pregnant mice at G16 and G18. Relative to uninfected controls, infected mice exhibited enlarged labyrinth zones, reduced junction zones, and narrowed vascular spaces at both time points, with more pronounced alterations at G18 (Figure S2) ” (Lines 386-390).

2.Lines: 407-410: statistical analysis should be shown for Supp Table 2 to support claims of “changes” in gut microbiota alpha diversity metrics.

Response: Thank you for your review. We have added the statistical analysis for the comparisons of gut microbiota alpha diversity metrics in Supplementary Table 2. The updated Table S2 has been uploaded.

3.Fig 3: PbD3 (G16) and PbD5 (G18) data should be compared against infected mice at the same infection time points to distinguish effects of (advancing) pregnancy from effects of infection. It is commendable that the authors generated data in nonpregnant infected animals; these data should be integrated and analyzed with the infected pregnant mouse data to support the claim in line 426-7.

Response: We thank the reviewer for this valuable suggestion. We agree that comparisons with infected non-pregnant mice at matched infection time points are important for distinguishing pregnancy-related effects from infection-induced microbiota alterations. In fact, these data had already been generated and included in the original supplementary dataset; however, we acknowledge that the corresponding description in the Results section was not sufficiently clear. Therefore, we have now revised the manuscript to explicitly present this comparison and clarify its interpretation (Supplementary Figure S3).

Result:“To exclude the potential effect of pregnancy on gut microbiota composition, we additionally analyzed infected non-pregnant mice at the corresponding infection time points (non-pregnant PbD3 and non-pregnant PbD5). Compared with infected non-pregnant mice, pregnant infected mice exhibited no significant differences in the relative abundance of the major bacterial taxa at either the phylum or genus level (Supplementary Figure S3). Specifically, neither Firmicutes nor Bacteroidota at the phylum level, nor representative genera including Ligilactobacillus, Lactobacillus, Akkermansia, and Desulfovibrio, differed significantly between the corresponding pregnant and non-pregnant infected groups (all P > 0.05). Collectively, these results suggest that pregnancy status did not substantially influence the core microbiota alterations associated with PbA infection”(Lines 441-451).

4.The analyses in Fig 5 need to correct for multiple hypothesis testing. Adjust P values with Holm-Sidak or similar.

Response: Thank you for your valuable comment. Following your suggestion, we performed multiple hypothesis testing correction for the microbiota–metabolite correlation analysis using the Holm–Šídák method and updated the Methods and Results sections accordingly. The adjusted P values are reported throughout the manuscript. The revised text is as follows:

“To explore the associations between differential bacterial genera and metabolites, Spearman's correlation analysis was performed and visualized as a heatmap (Figure 5A). Notably, the relative abundance of Monoglobus was positively correlated with LPC(22:6) (r=0.89, adjusted P < 0.05), suggesting a potential role for this genus in maintaining lipid metabolic homeostasis during infection. In contrast, the potentially pathogenic genus Desulfovibrio exhibited a strong negative correlation with 2-(2-(19-Acetamido-16,18-dihydroxy-5,9-dimethyl-6-oxoicosan-7-yl) oxy-2-oxoethyl) butanedioic acid (r=-0.86, adjusted P < 0.05), implying that the expansion of Desulfovibrio may contribute to the depletion of this metabolite during disease progression. In addition, Alistipes and Odoribacter respectively exhibited significant positive and negative correlations with multiple metabolites involved in lipid and nucleoside metabolism (|r| = 0.84-0.87, adjusted P < 0.05). Collectively, these results revealed significant associations between differential gut bacterial genera and host metabolites following PbA infection, highlighting coordinated alterations in the gut microbiota and metabolome.”(Lines488-502).

Discussion “Correlation analysis further demonstrated significant associations between altered bacterial genera and host metabolites involved in lipid and amino acid metabolism, highlighting coordinated changes in the gut microbiota and metabolome during PbA infection. ” (Lines 585-588).

5.What was the impact of FMT in the IN mice on the gut microbiota (Fig 7 and 8)? It is understandable if this was not measured, but it should be cited as a limitation of the study.

Response: We thank the reviewer for this comment. We acknowledge that the impact of FMT on gut microbiota composition in IN group was not directly assessed in this study, which represents a limitation.

We have added the limitation: “Thirdly, gut microbiota profile following FMT was not systematically characterized. Future studies are required to determine the impact of FMT on gut microbiota..” (Lines 625-626).

Part III – Minor Issues: Editorial and Data Presentation Modifications
Please use this section for editorial suggestions as well as relatively minor modifications of existing data that would enhance clarity.

Reviewer #1:

1. While I appreciate the broad title that does not make overstatements, the manuscript would benefit from a title that is a bit more specific to the reported outcomes.

Response: Thank you for your suggestion. We agree that a more specific title better reflects the main findings of our study. Accordingly, we have revised the title to:

“Fecal Microbiota Transplantation Alleviates Adverse Pregnancy Outcomes and Intestinal Injury in Experimental Malaria.”

2. Lines 36-38, 58-59, 552-554, 593-595 – I still have substantial concerns and find it very speculative to suggest that FMT will have a place in the clinical management and prevention of pregnancy malaria in malaria-endemic countries. Line 58-59 is reasonable with the exclusion of FMT.

Response: Thank the reviewer for this insightful comment. We agree that it very speculative to suggest that FMT will have a place in the clinical management and prevention of pregnancy malaria in malaria-endemic countries..

Accordingly, we have revised the manuscript and deleted the statements implying clinical applicability of FMT. The text in Lines 36–38, 58–59, 552–554, and 593–595 has been updated to ensure a more cautious and appropriate interpretation of our findings.

Lines 36–38: We had deleted “highlight the potential of FMT as a experimental strategy for managing malaria in pregnancy.”

Lines 58–59: we have removed the phrase “like FMT”. The sentence now:

”Our work uncovers a key mechanism linking the gut microbiome to pregnancy complications in malaria and points to microbiome-targeted strategies as potential future avenues for intervention.”

Lines 552–554: We had deleted “FMT from healthy donors provided a promising therapeutic approach of microbiota-targeted interventions in treating malaria-related pathology during pregnancy.”

Lines 593–595: We had deleted “A randomized, double-blind, placebo-controlled design would be recommended to rigorously evaluate the efficacy and safety of FMT as an adjunct to standard antimalarial therapy.”

3. The call out of Figure 2D is out of sequence.

Response: We thank the reviewer for this careful observation. We have systematically reviewed all figure in the manuscript and corrected the sequencing for Figure 2D.

4. Rationale for investigating intestinal barrier integrity - The authors should reference prior publications that have demonstrated Plasmodium infections disrupt intestinal barrier integrity (there are many). If not in the results, these should be included in the Introduction or Discussion sections.

Response: Thank you for this suggestion. We have added the relevant references and sentence in the Introduction section as follows:“Accumulating evidence indicates that Plasmodium infections disrupt intestinal barrier integrity through microvascular sequestration of infected erythrocytes, local inflammatory activation, and subsequent impairment of epithelial tight junctions (7-11). ” (Lines 86-89).

References

[7]Sriboonvorakul N, Chotivanich K, Silachamroon U, Phumratanaprapin W, Adams JH, Dondorp AM, et al. Intestinal injury and the gut microbiota in patients with Plasmodium falciparum malaria. PLoS Pathog. 2023;19(10):e1011661.

[8]Romero M, Leiba E, Carrión-Nessi FS, Freitas-De Nobrega DC, Kaid-Bay S, Gamardo Á F, et al. Malaria in pregnancy complications in Southern Venezuela. Malar J. 2021;20(1):186.

[9]Guida Marascia F, Colomba C, Abbott M, Gizzi A, Anastasia A, Pipitò L, et al. Imported malaria in pregnancy in Europe: A systematic review of the literature of the last 25 years. Travel Med Infect Dis. 2023;56:102673.

[10]Denny JE, Powers JB, Castro HF, et al. Differential Sensitivity to Plasmodium yoelii Infection in C57BL/6 Mice Impacts Gut-Liver Axis Homeostasis. Sci Rep. 2019;9(1):3472. Published 2019 Mar 5. doi:10.1038/s41598-019-40266-6

[11]Guan W, Yang S, Zhao Y, et al. Observation of the Gut Microbiota Profile in C57BL/6 Mice Induced by Plasmodium berghei ANKA Infection. Front Cell Infect Microbiol. 2021;11:680383. Published 2021 Oct 28. doi:10.3389/fcimb.2021.680383

5. Figure 4A – as I understand the Venn Diagram, the 109 metabolites are shared across the three groups, not “altered”. I think “altered” needs to be removed from this sentence.

Response: Thank you for your careful reading and suggestion. We had removed “alter”from this sentence.

6. The call out for Figure 7D is out of sequence.

Response: We thank the reviewer for their careful attention to detail. We have corrected the out-of-sequence callout for Figure 7D in the revised manuscript. All figure citations have now been verified and appear in the correct order.

Reviewer #2:

1.English language grammar and syntax need to be assessed and corrected throughout the manuscript.

Response: We thank the reviewer for this suggestion. The manuscript has undergone thorough language editing by a native English speaker to correct grammar and syntax throughout. We believe the readability has been significantly improved.

2.Introduction: Despite the authors’ claim, ref 2 does examine fetal outcomes; these results should be considered.

Response: Thank the reviewer for this important comment. We agree that reference 2 does examine fetal outcomes, and we apologize for the lack of clarity in our original description. In the revised manuscript, we have updated as:

“Smith et al. (2) demonstrated that gut microbiota composition was associated with both susceptibility to malaria during pregnancy and fetal outcomes, and further identified specific microbial taxa related to infection outcomes”(Line 105-108)

3.Line 400: Day 5 of a rodent malaria infection is not considered to be chronic.

Response: Thank the reviewer for this important comment. We agree that day 5 of *Plasmodium berghei* ANKA infection is not considered chronic phase in rodent malaria models. We have deleted “chronic” and revised “Taken together, these results suggest that PbA infection disrupts the intestinal barrier. ”

4.Figure 4: while it is agreed that the data shown do reflect changes across the three groups, the authors need to interpret their data carefully. As mentioned for the previous figures, the changes could be attributable to two factors in these groups: infection day and gestation day. The description and interpretation of the data must acknowledge this issue.

Response：Thank you for this important comment. We agree that the metabolic differences observed among the PbD0, PbD3, and PbD5 groups may reflect the combined influence of infection progression and gestational advancement because these variables change simultaneously in our experimental design. Accordingly, we have revised the Results section to avoid attributing all metabolic alterations solely to PbA infection and now describe these findings as differences among the experimental groups. We have also added a statement acknowledging that both infection progression and gestational stage may contribute to the observed metabolic profiles. In addition, we have further acknowledged this limitation in the Discussion.

Results: “Considering the concurrent effects of infection progression and advancing gestation, the observed metabolic alterations should be interpreted as reflecting the combined influence of both factors rather than being attributed solely to PbA infection. In addition, hierarchical clustering and volcano plot analyses further demonstrated distinct metabolic profiles among the three groups (Figure 4C and 4D).”( Line 470-475)

Discussion: “In addition, the observed microbial and metabolic alterations may be affected by infection progression and gestational advancement. Future studies incorporating larger cohorts and gestational age-matched controls are required, further to distinguish the effects of infection from those of pregnancy.” (Line 617-620)

5.Line 526-527: clarify, as the panel shows postnatal weight gain not fetal weight.

Response：We agree that Figure 8C reflects postnatal body weight rather than fetal weight. We have revised the manuscript: The results showed that offspring from the IN group exhibited significantly lower postnatal weight compared to the other two groups (Figure 8C).

6.Line 530: indicates “re-infection”. Were these mice infected prior to that shown in Figure 8D?

Response: We apologize for the confusion caused by the use of the term”re-infection”. In our experimental design, the offspring were not infected before birth. They were born to infected dams and subsequently subjected to their first PbA infection after 20 days after weaning.

We have revised: “After weaning at postnatal day 20, offspring were infected with PbA, and their survival was subsequently monitored (Figure 8D). ”
