# Supplementary material for: Fecal microbiota transplantation alleviates adverse pregnancy outcomes and intestinal injury in experimental malaria
Source: PLoS Pathog. 2026 Aug 20;22(8):e1014459. doi: 10.1371/journal.ppat.1014459 (PMC13492792; doi:10.1371/journal.ppat.1014459)
Supplement: Peer review history

# Peer review: Decision Letter 0

**DOI:** 10.1371/journal.ppat.1014459.r001

**Authors:** Margaret Phillips, Kevin Couper

7 Jun 2025

Gut Microbiota Modulation in Malaria-Infected Pregnant mice: Implications for Maternal and Neonatal Health

PLOS Pathogens

Dear Dr. Han,

Thank you for submitting your manuscript to PLOS Pathogens. After careful consideration, we feel that it has merit but does not fully meet PLOS Pathogens's publication criteria as it currently stands. Therefore, we invite you to submit a revised version of the manuscript that addresses the points raised during the review process.

Please submit your revised manuscript within 60 days Aug 06 2025 11:59PM. If you will need more time than this to complete your revisions, please reply to this message or contact the journal office at plospathogens@plos.org. When you're ready to submit your revision, log on to https://www.editorialmanager.com/ppathogens/ and select the 'Submissions Needing Revision' folder to locate your manuscript file.

Please include the following items when submitting your revised manuscript:

* A rebuttal letter that responds to each point raised by the editor and reviewer(s). You should upload this letter as a separate file labeled 'Response to Reviewers'. This file does not need to include responses to any formatting updates and technical items listed in the 'Journal Requirements' section below.

* A marked-up copy of your manuscript that highlights changes made to the original version. You should upload this as a separate file labeled 'Revised Manuscript with Track Changes'.

* An unmarked version of your revised paper without tracked changes. You should upload this as a separate file labeled 'Manuscript'.

If you would like to make changes to your financial disclosure, competing interests statement, or data availability statement, please make these updates within the submission form at the time of resubmission. Guidelines for resubmitting your figure files are available below the reviewer comments at the end of this letter.

We look forward to receiving your revised manuscript.

Kind regards,

Kevin N. Couper

Guest Editor

PLOS Pathogens

Margaret Phillips

Section Editor

Editor-in-Chief

PLOS Pathogens

PLOS Pathogens

orcid.org/0000-0002-7699-2064

**Additional Editor Comments:**

The reviewers have requested clarity on methodological details within the manuscript and they have requested additional mechanistic analyses be performed to improve the depth of investigations.  They have also requested that statistical approaches are re-evaluated.  Please attempt to respond to these comments when revising the manuscript.

**Journal Requirements:**

1) Please ensure that the CRediT author contributions listed for every co-author are completed accurately and in full.

At this stage, the following Authors/Authors require contributions: Su Han, Jie Wan, Weizhong Kong, Yu Zhang, Zhenli Xu, Rui Chen, Yang Cheng, and Yifan Sun. Please ensure that the full contributions of each author are acknowledged in the "Add/Edit/Remove Authors" section of our submission form.

The list of CRediT author contributions may be found here: https://journals.plos.org/plospathogens/s/authorship#loc-author-contributions

2) Please provide an Author Summary. This should appear in your manuscript between the Abstract (if applicable) and the Introduction, and should be 150-200 words long. The aim should be to make your findings accessible to a wide audience that includes both scientists and non-scientists. Sample summaries can be found on our website under Submission Guidelines:

https://journals.plos.org/plospathogens/s/submission-guidelines#loc-parts-of-a-submission

3) We do not publish any copyright or trademark symbols that usually accompany proprietary names, eg ©,  ®, or TM  (e.g. next to drug or reagent names). Therefore please remove all instances of trademark/copyright symbols throughout the text, including:

- ® on page: 9.

4) Please upload all main figures as separate Figure files in .tif or .eps format. For more information about how to convert and format your figure files please see our guidelines:

https://journals.plos.org/plospathogens/s/figures

5) We notice that your supplementary Figures are included in the manuscript file. Please remove them and upload them with the file type 'Supporting Information'. Please ensure that each Supporting Information file has a legend listed in the manuscript after the references list.

6) Some material included in your submission may be copyrighted. According to PLOSu2019s copyright policy, authors who use figures or other material (e.g., graphics, clipart, maps) from another author or copyright holder must demonstrate or obtain permission to publish this material under the Creative Commons Attribution 4.0 International (CC BY 4.0) License used by PLOS journals. Please closely review the details of PLOSu2019s copyright requirements here: PLOS Licenses and Copyright. If you need to request permissions from a copyright holder, you may use PLOS's Copyright Content Permission form.

Please respond directly to this email and provide any known details concerning your material's license terms and permissions required for reuse, even if you have not yet obtained copyright permissions or are unsure of your material's copyright compatibility. Once you have responded and addressed all other outstanding technical requirements, you may resubmit your manuscript within Editorial Manager.

Potential Copyright Issues:

i) Figure 1A. Please confirm whether you drew the images / clip-art within the figure panels by hand. If you did not draw the images, please provide (a) a link to the source of the images or icons and their license / terms of use; or (b) written permission from the copyright holder to publish the images or icons under our CC BY 4.0 license. Alternatively, you may replace the images with open source alternatives. See these open source resources you may use to replace images / clip-art:

- https://commons.wikimedia.org

- https://openclipart.org/.

7) Please note that your Data Availability Statement is currently include incorrect accession number of each dataset and missing a direct link to access each dataset. If your manuscript is accepted for publication, you will be asked to provide these details on a very short timeline. We therefore suggest that you provide this information now, though we will not hold up the peer review process if you are unable.

8) In the online submission form, you indicated that The data that support the findings of this study are available from the corresponding author, Su Han, upon reasonable request.. All PLOS journals now require all data underlying the findings described in their manuscript to be freely available to other researchers, either

1. In a public repository

2. Within the manuscript itself

3. Uploaded as supplementary information.

This policy applies to all data except where public deposition would breach compliance with the protocol approved by your research ethics board. If your data cannot be made publicly available for ethical or legal reasons (e.g., public availability would compromise patient privacy), please explain your reasons by return email and your exemption request will be escalated to the editor for approval. Your exemption request will be handled independently and will not hold up the peer review process, but will need to be resolved should your manuscript be accepted for publication. One of the Editorial team will then be in touch if there are any issues.

**Reviewers' Comments:**

Reviewer's Responses to Questions

**Part I - Summary**

Please use this section to discuss strengths/weaknesses of study, novelty/significance, general execution and scholarship.

Reviewer #1: The article by Han S, et al., reports on the effect of P. berghei ANKA-induced changes on gut microbiota and the effect these have on pregnancy malaria outcomes. Specifically, the authors demonstrate that P. berghei ANKA causes changes in the gut microbiome that associates with changes in serum metabolites. Treatment of pregnant mice infected with P. berghei with antibiotics had no effect on fetal weight but decreased expression of claudin-1 and occluding with concomitant increase in the expression of IFN-g and TNF. They also report that fecal microbiota transplants from uninfected pregnant mice decreased P. berghei-induced intestinal damage and resulted in increased fetal weight compared to untreated P. berghei-infected pregnant mice. A major concern is the lack of mechanistic insight provided in this report especially as a prior report has described gut microbiota contributions towards pregnancy malaria outcomes (PMID: 31160271).

Reviewer #2: This manuscript explores the interactions between gut health and malaria infection in a pregnant mouse model. The experimental work is extensive and comprehensive, and shows clearly that infection impacts gut health and the microbiota, that removal of the gut microbiota with antibiotic treatment impacts gut integrity (without impacting fetal outcomes) and transfaunation of gut microbiota improves gut health, reduces IFN-gamma production, and improves postnatal growth of pups. More methodological detail, reassessment of statistical analyses and a broader consideration of the results will improve the manscript.

Reviewer #3: Strengths

The study employs a comprehensive approach, combining gut microbiota analysis, metabolomics, and fecal microbiota transplantation experiments to elucidate the role of gut microbiota in malaria-infected pregnant mice.

The use of multiple experimental groups and the inclusion of both early and late stages of infection help to provide a more complete picture of the impact of PbA infection on the gut-placenta axis.

Weaknesses

Clinical Relevance: While the study provides valuable insights into the role of gut microbiota in malaria-infected pregnant mice, the relevance of these findings to human malaria infection remains uncertain. The authors should acknowledge this limitation and suggest future studies to validate the findings in human populations.

Mechanistic Insights: The study demonstrates that FMT can alleviate placental and colonic inflammation and improve fetal weight, but the underlying mechanisms by which the gut microbiota influences these outcomes are not fully elucidated. Further exploration of the specific pathways and molecules involved would strengthen the study.

Experimental Design: The study does not include a control group of non-pregnant malaria-infected mice, which would have helped to distinguish the effects of pregnancy on the gut microbiota and placental pathology in the context of malaria infection.

Sample Size and Statistical Power: The sample size appears to be relatively small, which may limit the statistical power of the study and the generalizability of the findings. The authors should provide a justification for the chosen sample size and consider whether a larger sample size would be necessary for future studies.

Data Analysis: The analysis of the gut microbiota composition and metabolomics data is comprehensive, but the integration of these datasets could be improved. A more detailed analysis of the relationships between specific gut microbiota changes and metabolite alterations would provide a clearer understanding of the mechanisms involved.

**********

**Part II – Major Issues: Key Experiments Required for Acceptance**

Please use this section to detail the key new experiments or modifications of existing experiments that should be absolutely required to validate study conclusions.

Generally, there should be no more than 3 such required experiments or major modifications for a "Major Revision" recommendation. If more than 3 experiments are necessary to validate the study conclusions, then you are encouraged to recommend "Reject".

Reviewer #1: There are several occasions in which the authors overstate their conclusions. These include but are not limited to the following:

o Line 39-41 – Overstatement to suggest FMT holds promise as strategy for managing pregnancy malaria.

o Line 415 – Data do not evaluate pregnancy outcomes

o Line 439 – “notable colonic inflammation” is overstated as only IFN-g is different.

Line 47-48 – Recommend using updated numbers from the WHO.

Introduction – The authors have failed to provide an appropriate summary of the previous work by Ref 2 in regards to gut microbiota-pregnancy malaria outcomes in mice.

Figure 1, 6, and 7 – the authors should demonstrate that the changes in tight junction proteins results in decreased intestinal barrier integrity. One approach would be to gavage mice with FITC-dextran and assess FITC-dextral levels in the circulations.

Figure 6.

o Details of antibiotic treatment days are not clear nor when the mice were harvested for subsequent analysis.

o There is no evidence that antibiotic treatment altered gut bacteria abundance or composition.

o Not clear how the antibiotic treatment in which most gut bacteria are eliminated is informative towards P. berghei ANKA-induced disruptions in gut bacteria on pregnancy outcomes.

Figure 7 – the experiment would benefit from a vehicle control and gut microbiota analysis to demonstrate that the FMT changed the gut microbiota composition of treated animals.

Figure 8

o Line 459-460 – were pups infected with P. berghei? The experimental plan of this data set is not clear.

o No statistics are provided for panel C or panel D.

o Experimental set up is not clear and data do not make sense. It seems shocking that no pups survive beyond day 11 in the untreated group. Additionally, if no pups survive beyond day 11 in the untreated group, how is there weight measurement for this group through day 20?

Reviewer #2: The statistical analysis subsection of the methods section mentions statistical comparisons being done with students t test but this is not appropriate to analyses with three groups. Figure legends should specify statistical tests applied.

Does Fig 5 correct for false discovery rate/multiple hypothesis testing?

Statements at lines 409-416 are not fully supported by the data shown in Fig 6.

Without more details regarding the timing of ABX treatment/transfaunation/FMT, it is difficult for the reader to interpret Figure 7.

Individual fetus weights are not independent, as they are influenced by the intrauterine environment, including number of total fetuses present (per dam). Statistical analysis (Fig 8B) must take into account this lack of independence.

The manuscript provides no methodological details of the data shown in Figure 8D-F.

Reviewer #3: What metabolites have undergone significant changes and what roles do they play?

**********

**Part III – Minor Issues: Editorial and Data Presentation Modifications**

Please use this section for editorial suggestions as well as relatively minor modifications of existing data that would enhance clarity.

Reviewer #1: Grammer and sentence structure should be carefully reviewed throughout the text.

Reviewer #2: Methods

Provide more details regarding how fostering of newborn pups to surrogate dams was performed.

How was gut microbial depletion confirmed? What was the timing used for ABX treatment and transfaunation of gut microbiota in relation to initiation of pregnancy and PbA infection? Were FMT mice ABX treated first or just received an FMT? Was the FMT material from pregnant mice?

Results

Figure resolution is low throughout, making it difficult to evaluate.

Line 303: provide reference(s)

Samples sizes included in each figure/panel should be provided in the legend unless it is obvious (ie Fig 3 scatter plots or individual microbiota compositions).

Check typo at lines 346-7, re Fig 3E

Lines 348-350: why are only two of the highlighted genera shown in Figure 3F?

Section starting at line 351: Not sure it is appropriate to refer to differentially abundant genera as “biomarkers”

What are the y axis units for Fig 4G?

Statement at lines 248-430 is not supported by the data show in Fig 7.

Figure 7D requires more annotation to support the statement made at lines 431-433.

The data shown in Fig S1 are important and their incorporation into the body of the manuscript is recommended.

Discussion

The results with ABX and FMT were mixed (less inflammation but reduced crypt length with FMT; with ABX alone, reduced tight junction protein expression but increased inflammation). More discussion is required to interpret these results.

Line 509: what is meant by “prolonged” PbA infection?

Reviewer #3: Title and Abstract:

The title is appropriate and concise, accurately reflecting the core content of the study. However, the abstract should better highlight the innovative aspects of the gut microbiome research in malaria-infected pregnant mice and its clinical implications. For example, the specific changes in gut microbiota and their direct impact on pregnancy outcomes could be emphasized.

Introduction:

The background introduction is comprehensive, but it could more clearly establish the connection between gut microbiota and malaria infection in pregnant mice. The current explanation remain unclear. For instance, when discussing the impact of malaria on pregnant women, it could specifically mention how these effects are linked to gut microbiota alterations. For Malaria Data, please update to 2023.

The literature review could be more focused. While citing numerous studies on malaria and gut microbiota, it should better integrate them with the current research objectives. For example, after introducing previous findings on gut microbiota changes in malaria patients, it should explicitly state the gaps these studies leave and how this study addresses them.

Methods:

In the animal experiment section, the grouping and infection procedures are clearly described. However, the criteria for selecting the specific dose and timing of infection could be explained. For example, why was day 13 of gestation chosen for infection? What is the basis for using an intravenous inoculation of 1×106 PbA-infected erythrocytes? This information would help readers better understand the experimental design rationale.

For the FMT experiment, the preparation and transplantation process of fecal microbiota is detailed. Nevertheless, the potential risks and quality control measures of FMT, such as how to avoid the transmission of pathogens, could be mentioned to enhance the reliability of the experimental methods.

Results:

The results are rich in data, but the presentation could be more organized. For example, the histopathological changes of the placenta and colon, alterations in gut microbiota composition, and variations in serum metabolites could be grouped and discussed in a logical sequence to make it easier for readers to follow.

Some graphical representations of the results could be improved. For instance, in Figures 1–8, the labels and legends could be made clearer and more specific. In Figure 1, the representative images of H&E-stained placental tissue sections could indicate the specific regions being observed and the differences between groups. In Figure 2, the histopathological changes of colonic samples could provide a more detailed description of the inflammatory cell infiltration.

The correlation analysis between gut microbiota and serum metabolites is a key part of the results. However, the interpretation of the correlation coefficients and p-values in the correlation analysis results could be more in-depth. For example, what biological significance do the positive and negative correlations between specific gut microbiota and metabolites imply?

Discussion:

The discussion effectively links the results to the research objectives and hypotheses. However, it could more critically evaluate the limitations of the study. For example, apart from the limitation of using an animal model, other potential limitations such as the relatively small sample size and the specificity of the experimental conditions could be discussed.

When discussing the clinical implications of the findings, it could more explicitly suggest directions for future research. For instance, it could propose specific plans for clinical trials of FMT in pregnant women with malaria, including trial design, participant selection, and outcome indicators.

**********

PLOS authors have the option to publish the peer review history of their article (what does this mean?). If published, this will include your full peer review and any attached files.

If you choose “no”, your identity will remain anonymous but your review may still be made public.

**Do you want your identity to be public for this peer review?**  For information about this choice, including consent withdrawal, please see our Privacy Policy.

Reviewer #1: No

Reviewer #2: No

Reviewer #3: No

[NOTE: If reviewer comments were submitted as an attachment file, they will be attached to this email and accessible via the submission site. Please log into your account, locate the manuscript record, and check for the action link "View Attachments". If this link does not appear, there are no attachment files.]

**Figure resubmission:**

While revising your submission, please upload your figure files to the Preflight Analysis and Conversion Engine (PACE) digital diagnostic tool, https://pacev2.apexcovantage.com/. PACE helps ensure that figures meet PLOS requirements. To use PACE, you must first register as a user. Registration is free. Then, login and navigate to the UPLOAD tab, where you will find detailed instructions on how to use the tool. If you encounter any issues or have any questions when using PACE, please email PLOS at figures@plos.org. Please note that Supporting Information files do not need this step. If there are other versions of figure files still present in your submission file inventory at resubmission, please replace them with the PACE-processed versions.

**Reproducibility:**

?>

# Author comment: Author response to Decision Letter 1

**DOI:** 10.1371/journal.ppat.1014459.r002

9 Jan 2026

Attachment Submitted filename: LETTER 2025.12.24docx.docx

# Peer review: Decision Letter 1

**DOI:** 10.1371/journal.ppat.1014459.r003

**Authors:** Margaret Phillips, Kevin Couper

18 Feb 2026

PPATHOGENS-D-25-00948R1

Gut Microbiota Modulation in Malaria-Infected Pregnant mice: Implications for Maternal and Neonatal Health

PLOS Pathogens

Dear Dr. Han,

Thank you for submitting your manuscript to PLOS Pathogens. After careful consideration, we feel that it has merit but does not fully meet PLOS Pathogens's publication criteria as it currently stands. Therefore, we invite you to submit a revised version of the manuscript that addresses the points raised during the review process.

Please submit your revised manuscript by Apr 19 2026 11:59PM. If you will need more time than this to complete your revisions, please reply to this message or contact the journal office at plospathogens@plos.org. When you're ready to submit your revision, log on to https://www.editorialmanager.com/ppathogens/ and select the 'Submissions Needing Revision' folder to locate your manuscript file.

Please include the following items when submitting your revised manuscript:

* A letter that responds to each point raised by the editor and reviewer(s). You should upload this letter as a separate file labeled 'Response to Reviewers'. This file does not need to include responses to any formatting updates and technical items listed in the 'Journal Requirements' section below.

* A marked-up copy of your manuscript that highlights changes made to the original version. You should upload this as a separate file labeled 'Revised Manuscript with Track Changes'.

* An unmarked version of your revised paper without tracked changes. You should upload this as a separate file labeled 'Manuscript'.

If you would like to make changes to your financial disclosure, competing interests statement, or data availability statement, please make these updates within the submission form at the time of resubmission. Guidelines for resubmitting your figure files are available below the reviewer comments at the end of this letter.

We look forward to receiving your revised manuscript.

Kind regards,

Kevin N. Couper

Guest Editor

PLOS Pathogens

Margaret Phillips

Section Editor

PLOS Pathogens

Sumita Bhaduri-McIntosh

Editor-in-Chief

PLOS Pathogens

orcid.org/0000-0003-2946-9497

Michael Malim

Editor-in-Chief

PLOS Pathogens

orcid.org/0000-0002-7699-2064

**Additional Editor Comments:**

Both reviewers agree that the manuscript has been substantially improved; however, the reviewers still highlight some concerns with the manuscript. Please respond to the reviewer's comments, and in particular please address reviewer 2's comments on the statistical analyses and the integration of required data in figures 1 and 3 of the manuscript to distinguish effects of gestational age and infection.

**Journal Requirements:**

Please ensure that the CRediT author contributions listed for every co-author are completed accurately and in full.

At this stage, the following Authors/Authors require contributions: Su Han, Jie Wan, DAI RONGRONG, Weizhong Kong, Zhenli Xu, Yu Zhang, Rui Chen, Yang Cheng, and Yifan Sun. Please ensure that the full contributions of each author are acknowledged in the "Add/Edit/Remove Authors" section of our submission form.

The list of CRediT author contributions may be found here: https://journals.plos.org/plospathogens/s/authorship#loc-author-contributions

**Reviewers' Comments:**

Reviewer's Responses to Questions

**Part I - Summary**

Please use this section to discuss strengths/weaknesses of study, novelty/significance, general execution and scholarship.

Reviewer #1: Han S, et al, have provided an extensively revised manuscript that addresses many of my prior concerns. I find this to be substantially improved manuscript that will be an important contribution to the malaria-gut microbiome research community. I do have several remaining concerns.

Reviewer #2: This revised manuscript examines how malaria infection in a pregnant mouse model impacts the gut microbiota, intestinal integrity, metabolism and offspring outcomes. The revision presents an improved manuscript, but some additional revisions are required to meet journal standards.

Results

**********

**Part II – Major Issues: Key Experiments Required for Acceptance**

Please use this section to detail the key new experiments or modifications of existing experiments that should be absolutely required to validate study conclusions.

Generally, there should be no more than 3 such required experiments or major modifications for a "Major Revision" recommendation. If more than 3 experiments are necessary to validate the study conclusions, then you are encouraged to recommend "Reject".

Reviewer #1: 1. Line 499-494 – The data suggest a more nuanced interpretation than “While colonic damage and inflammation caused by PbA infection are dependent on the gut microbiota.” Following antibiotic-induced reduction in gut microbiota, there is improved intestinal barrier integrity (reduction in serum FITC-dextran) while reduced expression of claudin-1 and occluding. These latter results suggest reduced intestinal barrier integrity, which is consistent with increased pro-inflammatory cytokines, IFN-g and TNF. Instead of a broad, non-specific conclusion the authors can provide more specific interpretations and conclusions from these results.

Reviewer #2: No new experiments are requested. However, some data reanalysis should be considered.

Methods

Methods used for pathological and quantitative evaluation of placenta and intestinal tissue (data shown in Figure 1, 2, 7) need to be described. Were the samples evaluated in a blinded fashion? By how many independent evaluators?

Evaluation of fetal and postnatal outcomes: Is it correct that n=2 dams per group?

Statistics: Correlation analysis (Fig 5) must either control for multiple hypothesis testing with posthoc adjustments, or the limitations of failing to account for Type I error must be acknowledged in the methods and results sections. For groupwise comparisons, Kruskal-Wallis test was used with which posthoc test for pairwise comparisons?

Results

Figure 1A, B, C, D compare mice across different gestation days when placental morphology would be expected to change. PbD3 (G16) and PbD5 (G18) data should be compared against uninfected pregnant mice at the same gestational age. Lines 155-157 indicate such mice were generated; their data should be shown in Fig 1. Without these critical control data, the results are difficult to interpret as the effects of advancing pregnancy cannot be distinguished from the effects of advancing infection.

Lines: 407-410: statistical analysis should be shown for Supp Table 2 to support claims of “changes” in gut microbiota alpha diversity metrics.

Fig 3: PbD3 (G16) and PbD5 (G18) data should be compared against infected mice at the same infection time points to distinguish effects of (advancing) pregnancy from effects of infection. It is commendable that the authors generated data in nonpregnant infected animals; these data should be integrated and analyzed with the infected pregnant mouse data to support the claim in line 426-7.

The analyses in Fig 5 need to correct for multiple hypothesis testing. Adjust P values with Holm-Sidak or similar.

What was the impact of FMT in the IN mice on the gut microbiota (Fig 7 and 8)? It is understandable if this was not measured, but it should be cited as a limitation of the study.

**********

**Part III – Minor Issues: Editorial and Data Presentation Modifications**

Please use this section for editorial suggestions as well as relatively minor modifications of existing data that would enhance clarity.

Reviewer #1: 1. While I appreciate the broad title that does not make overstatements, the manuscript would benefit from a title that is a bit more specific to the reported outcomes.

2. Lines 36-38, 58-59, 552-554, 593-595 – I still have substantial concerns and find it very speculative to suggest that FMT will have a place in the clinical management and prevention of pregnancy malaria in malaria-endemic countries. Line 58-59 is reasonable with the exclusion of FMT.

3. The call out of Figure 2D is out of sequence.

4. Rationale for investigating intestinal barrier integrity - The authors should reference prior publications that have demonstrated Plasmodium infections disrupt intestinal barrier integrity (there are many). If not in the results, these should be included in the Introduction or Discussion sections.

5. Figure 4A – as I understand the Venn Diagram, the 109 metabolites are shared across the three groups, not “altered”. I think “altered” needs to be removed from this sentence.

6. The call out for Figure 7D is out of sequence.

Reviewer #2: English language grammar and syntax need to be assessed and corrected throughout the manuscript.

Introduction: Despite the authors’ claim, ref 2 does examine fetal outcomes; these results should be considered.

Line 400: Day 5 of a rodent malaria infection is not considered to be chronic.

Figure 4: while it is agreed that the data shown do reflect changes across the three groups, the authors need to interpret their data carefully. As mentioned for the previous figures, the changes could be attributable to two factors in these groups: infection day and gestation day. The description and interpretation of the data must acknowledge this issue.

Line 526-527: clarify, as the panel shows postnatal weight gain not fetal weight.

Line 530: indicates “re-infection”. Were these mice infected prior to that shown in Figure 8D?

**********

PLOS authors have the option to publish the peer review history of their article (what does this mean?). If published, this will include your full peer review and any attached files.

If you choose “no”, your identity will remain anonymous but your review may still be made public.

**Do you want your identity to be public for this peer review?**  For information about this choice, including consent withdrawal, please see our Privacy Policy.

Reviewer #1: No

Reviewer #2: No

[NOTE: If reviewer comments were submitted as an attachment file, they will be attached to this email and accessible via the submission site. Please log into your account, locate the manuscript record, and check for the action link "View Attachments". If this link does not appear, there are no attachment files.]

**Figure resubmission:**

**Reproducibility:**

To enhance the reproducibility of your results, we recommend that authors of applicable studies deposit laboratory protocols in protocols.io, where a protocol can be assigned its own identifier (DOI) such that it can be cited independently in the future. Additionally, PLOS ONE offers an option to publish peer-reviewed clinical study protocols. Read more information on sharing protocols at https://plos.org/protocols?utm_medium=editorial-email&utm_source=authorletters&utm_campaign=protocols

# Author comment: Author response to Decision Letter 2

**DOI:** 10.1371/journal.ppat.1014459.r004

2 Jul 2026

Attachment Submitted filename: Response to Reviewers 2026.0630.docx

# Editor report: Decision Letter 2

**DOI:** 10.1371/journal.ppat.1014459.r005

**Authors:** Margaret Phillips, Kevin Couper

8 Jul 2026

Dear professor Han,

We are pleased to inform you that your manuscript 'Fecal Microbiota Transplantation Alleviates Adverse Pregnancy Outcomes and Intestinal Injury in Experimental Malaria' has been provisionally accepted for publication in PLOS Pathogens.

Before your manuscript can be formally accepted you will need to complete some formatting changes, which you will receive in a follow up email. A member of our team will be in touch with a set of requests.

Please note that your manuscript will not be scheduled for publication until you have made the required changes, so a swift response is appreciated.

IMPORTANT: The editorial review process is now complete. PLOS will only permit corrections to spelling, formatting or significant scientific errors from this point onwards. Requests for major changes, or any which affect the scientific understanding of your work, will cause delays to the publication date of your manuscript.

Should you, your institution's press office or the journal office choose to press release your paper, you will automatically be opted out of early publication. We ask that you notify us now if you or your institution is planning to press release the article. All press must be co-ordinated with PLOS.

Thank you again for supporting Open Access publishing; we are looking forward to publishing your work in PLOS Pathogens.

Best regards,

Kevin N. Couper

Guest Editor

PLOS Pathogens

Margaret Phillips

Section Editor

PLOS Pathogens

Sumita Bhaduri-McIntosh

Editor-in-Chief

PLOS Pathogens

orcid.org/0000-0003-2946-9497

Michael Malim

Editor-in-Chief

PLOS Pathogens

orcid.org/0000-0002-7699-2064

***********************************************************

Reviewer Comments (if any, and for reference):

# Editor report: Acceptance letter

**DOI:** 10.1371/journal.ppat.1014459.r006

**Authors:** Margaret Phillips, Kevin Couper

Dear professor Han,

We are delighted to inform you that your manuscript, "Fecal Microbiota Transplantation Alleviates Adverse Pregnancy Outcomes and Intestinal Injury in Experimental Malaria," has been formally accepted for publication in PLOS Pathogens.

We have now passed your article onto the PLOS Production Department who will complete the rest of the pre-publication process. All authors will receive a confirmation email upon publication.

The corresponding author will soon be receiving a typeset proof for review, to ensure errors have not been introduced during production. Please review the PDF proof of your manuscript carefully, as this is the last chance to correct any scientific or type-setting errors. Please note that major changes, or those which affect the scientific understanding of the work, will likely cause delays to the publication date of your manuscript. Note: Proofs for Front Matter articles (Pearls, Reviews, Opinions, etc...) are generated on a different schedule and may not be made available as quickly.

Soon after your final files are uploaded, the early version of your manuscript, if you opted to have an early version of your article, will be published online. The date of the early version will be your article's publication date. The final article will be published to the same URL, and all versions of the paper will be accessible to readers.

For Research Articles, you will receive an invoice from PLOS for your publication fee after your manuscript has reached the completed accept phase. If you receive an email requesting payment before acceptance or for any other service, this may be a phishing scheme. Learn how to identify phishing emails and protect your accounts at https://explore.plos.org/phishing.

Thank you again for supporting open-access publishing; we are looking forward to publishing your work in PLOS Pathogens.

Best regards,

Sumita Bhaduri-McIntosh

Editor-in-Chief

PLOS Pathogens

orcid.org/0000-0003-2946-9497

Michael Malim

Editor-in-Chief

PLOS Pathogens

orcid.org/0000-0002-7699-2064
